# Electrochemical Removal of Cephalosporin Antibiotic—Cefuroxime Axetil from Aquatic Media Using Boron-Doped Diamond Electrodes: Process Optimization, Degradation Studies and Transformation Products Characterization

**DOI:** 10.3390/molecules31010106

**Published:** 2025-12-26

**Authors:** Michał Wroński, Jakub Trawiński, Robert Skibiński

**Affiliations:** Department of Medicinal Chemistry, Faculty of Pharmacy, Medical University of Lublin, Jaczewskiego 4, 20-090 Lublin, Poland

**Keywords:** antibiotic drugs, BDD electrode, wastewater treatment, UHPLC-Q-TOF, TOC

## Abstract

Growing environmental concern over pharmaceutical contaminants in water, combined with the limited effectiveness of conventional treatment methods in removing persistent antibiotics, creates a need for advanced remediation technologies. This study investigates the degradation of the cephalosporin antibiotic cefuroxime axetil using an electrochemical advanced oxidation process with a boron-doped diamond (BDD) anode. Experiments were conducted under varying pH levels and in natural water matrices, specifically river and lake water, to evaluate the process efficiency under realistic conditions. Significant differences were observed between matrices, with the best result obtained in river water, enabling complete degradation of cefuroxime axetil within 30 min. To clarify the factors influencing process efficiency, additional experiments examined the effects of dissolved organic matter (DOM) and chlorides. Cefuroxime axetil proved highly susceptible to electrooxidation, generally following pseudo-first-order kinetics, and chloride significantly accelerated its degradation. Using high-resolution mass spectrometry, ten transformation products were identified, including six not previously reported in the literature, representing a key novelty of this work. Their potential aquatic toxicity was subsequently evaluated in silico using fish and algae models. Finally, energy consumption analysis was conducted to evaluate the impact of various factors on the process’s economic efficiency.

## 1. Introduction

Cefuroxime axetil is a ß-lactam antibiotic and a second-generation cephalosporin administered orally as a prodrug. Its esterified structure increases lipophilicity, enhancing gastrointestinal absorption. Once absorbed, it undergoes rapid hydrolysis and de-esterification in intestinal mucosa, releasing the active compound, cefuroxime, which is primarily excreted in this form [1]. Cefuroxime exerts a broad-spectrum bactericidal activity against Gram-positive and Gram-negative bacteria by binding to penicillin-binding proteins (PBPs), thereby inhibiting bacterial cell wall synthesis [2].

Cefuroxime axetil exists as a mixture of A and B diastereoisomers (Figure S1), whose stereochemistry influences its bactericidal actibity [1,2]. The syn-geometric isomer (Z-isomer) shows higher resistance to β-lactamase enzymes, whereas the anti-geometric isomer (E-isomer) is more susceptible to deactivation by cephalosporinases. Anti-cefuroxime axetil diastereoisomers (A and B epimers) and Δ3-cefuroxime axetil are known to occur as related impurities and potential degradation products of cefuroxime axetil [1]. Exposure to ultraviolet light, elevated temperatures, or acidic conditions can lead to the formation of anti-cefuroxime axetil as a degradation product.

The release of antibiotic pharmaceuticals into the environment can directly contribute to the development and spread of bacterial resistance. The emergence of antibiotic-resistant bacteria poses a serious public health risk, making infections harder to treat and reducing the effectiveness of existing therapies. Apart from contributing to antibiotic resistance, pharmaceuticals and their transformation products (TPs) can also exert toxic effects on non-target organisms. Żandarek et al. demonstrated through in vitro and in vivo studies that the photodegradation products of cefuroxime axetil exhibit greater toxicity than the parent compound alone [3]. Moreover, the overall toxicity of the mixture of cefuroxime axetil and its photoproducts increases the longer exposure lasts.

Due to its widespread use and broad range of applications, cefuroxime residues are frequently detected in various aquatic compartments, including wastewater, surface water, and groundwater, posing significant threats to non-target organisms. The primary sources of environmental antibiotic contamination include human excretion following administration, veterinary use, and improper disposal of expired medications. Numerous studies over the past decade have reported the presence of cefuroxime in environmental matrices worldwide. For example, Mutiyar and Mittal found that conventional sewage treatment plant do not fully remove cefuroxime, detecting concentration as high as 1.7 μg L^−1^ in the Yamuna river in northern India [4]. In Egypt, the compound was found in wastewater treatment plant at 2.1 ng L^−1^ [5]. It was also present in the effluents of pharmaceutical plants in Vietnam [6], wastewater treatment plant effluents in Germany [7] and China [8], as well as in hospital wastewater in China [9]. In contrast, Azanu et al. found cefuroxime in surface water (vegetable irrigation water) and lettuce samples from farming land and markets in Ghana [10,11]. Barreiro et al. found cefuroxime in soil samples under corn, with maximum values of 276 ng g^−1^ [12].

The variety of methods for treating effluents contaminated with cefuroxime have been already established to minimize surface water contamination and the formation of even more toxic byproducts. It is important to note that the majority of these studies have focused on cefuroxime in its active form, whereas cefuroxime axetil, the orally administered prodrug, has received relatively little attention. The explored methods include, among other, removal by adsorbents [13,14], bioremediation [15,16], photolytic ozonation [17], biocatalysis [18], or photocatalysis [19,20,21,22]. In addition to these, electrochemical methods represent a promising technology for degrading pollutants in wastewater. By employing oxidation and reduction reactions, they effectively break down a wide range of organic compounds and metal contaminants [23]. Harmful substances undergo complete mineralization into non-toxic end products such as carbon dioxide and water, or, alternatively, are transformed into less toxic and more biodegradable intermediates. One of the key advantages of electrooxidation lies in its environmentally friendly approach: it generates highly reactive hydroxyl radicals (•OH) directly at the electrode surface, eliminating the need for external chemical oxidants. This makes it a clean and efficient option for modern wastewater treatment applications.

In the study by Haji et al., an integrated approach combining homogeneous electro-Fenton pretreatment using a platinum anode with subsequent biological treatment using activated sludge was developed to effectively degrade and mineralize cefuroxime sodium in aqueous solutions [23]. In contrast, Chen and Hu proposed a method for the degradation of cefuroxime by integrating biochemical and electrochemical processes, employing graphite plate electrodes as the electrode material [24]. In the method developed by Yang et al., cefuroxime sodium wastewater was treated with a redox flow fuel cell utilizing a photothermal effect and FeCl_3_ as a photocatalyst and electron carrier to enhance degradation while generating electricity [25].

Tutunaru and Oprea studied the influence of pH and halide ions on cefuroxime electrochemical degradation with Pt/Pt system [26]. The best results were obtained under acidic conditions when bromide solution was used, outperforming both chloride and fluoride environments. Kurt et al. investigated the degradation of cefuroxime using Sb–SnO_2_/Ti anodes and demonstrated that pH played a crucial role in removal efficiency, with neutral pH yielding the best results [27].

The choice of electrode is crucial to ensure the feasibility of the system. Among the various anodes options, boron-doped diamond (BDD) stands out as the most extensively studied and successful electrode material for oxidizing organic molecules in various water matrices [28]. Its exceptional performance in real and simulated effluents is attributed to its ability to generate quasi-free hydroxyl radicals, high oxygen overvoltage, outstanding corrosion resistance in aggressive environments, and resistance to deactivation, making it the preferred choice for such applications. Recently, electrooxidation with BDD anode has been demonstrated as an effective approach for degrading the antibiotic of sulfamethoxazole [29].

However, the efficiency of this process can be significantly influenced by the chemical composition of the treated water. In real environmental and wastewater treatment scenarios, factors such as pH, concentrations of inorganic ions, and natural organic matter can affect the generation and activity of reactive oxidative species. Therefore, understanding how these factors influence the process is crucial for optimizing electrooxidation under realistic conditions. In this study, we investigated, for the first time, the electrochemical degradation of cefuroxime axetil using a BDD electrode, examining the influence of pH (neutral, acidic, and alkaline conditions) as well as the effect of real environmental matrices—river water and lake water—on the degradation efficiency. Additionally, the effects of chlorides and the presence of organic matrix components, using humic acid, fulvic acid, and natural organic matter at two concentration levels, were investigated to estimate the impact of varying environmental conditions. Formed TPs were identified by the means of high-resolution tandem mass spectrometry. Their toxic potential towards the aquatic species was preliminarily assessed using several in silico models. Finally, the degree of mineralization achieved during the studied processes was also evaluated.

## 2. Materials and Methods

### 2.1. Chemicals and Reagents

Cefuroxime axetil and sodium phosphate monobasic monohydrate salt were purchased from Sigma-Aldrich (St. Louis, MO, USA). Suwanee river humic (SRHA) acid, Suwanee river fulvic acid (SRFA) and Suwanee river natural organic matter (SRNOM) were purchased from International Humic Substances Society (St. Paul, MN, USA). Sodium phosphate dibasic anhydrous salt was obtained from J.T. Baker (Phillipsburg, NJ, USA). Acetonitrile and LC-MS grade water, as well as LC-grade water, were sourced from Witko (Łódź, Poland). Water for TOC analysis was purchased from Sigma-Aldrich (St. Louis, MO, USA). 98% formic acid (MS grade) and sodium chloride were obtained from Avantor Performance Materials Poland S.A. (Gliwice, Poland).

River water (RW) samples were collected from the Vistula River, upstream of Puławy city, and lake water (LW) were collected from a dystrophic (humic) lake in the Łęczna-Włodawa lake district (Western Polesia macro-region, eastern Poland). The parameters of the river water and lake water were measured and presented in the Table S1.

### 2.2. Electrochemical Experiments

The electrochemical degradation experiments were performed in galvanostatic mode using a Metrohm Autolab PGSTAT302N potentiostat/galvanostat (Utrecht, The Netherlands), operated with Nova 2.1.6. software. Boron-doped diamond (BDD) electrode, measuring 1 cm × 1 cm with an approximate electroactive surface area of 2 cm^2^, was employed as working electrode. A platinum coiled wire served as the auxiliary electrode. The electrodes were placed 0.6 cm apart at same depth. For each experiment, 20 mL of the solution was electrolyzed in a bulk electrolysis cell.

The stock solution of cefuroxime axetil was prepared by dissolving the compound in HPLC-grade synthetic water to obtain a concentration of 0.5 mg mL^−1^. All experiments were carried out at room temperature using a solution of cefuroxime axetil at the concentration 15 μg mL^−1^ in a 20 mM phosphate buffer, adjusted to pH 3, 7, or 9. All electrochemical treatments were performed in galvanostatic mode with a current intensity of 10 mA (5 mA cm^−2^).

During the experiments, various experimental parameters and their ranges were investigated, including pH levels (3, 7, and 9), the influence of river water and lake water matrices, chloride concentrations (50 and 100 mg L^−1^), and the impact of dissolved organic matter (DOM). The latter was evaluated by introducing humic acid (HA), fulvic acid (FA), and natural organic matter (NOM), which were tested at two concentrations levels of 10 and 20 mg L^−1^. All experiments with the addition of chloride and DOM were conducted in a phosphate buffer at pH 7. For experiments with DOM, a stock solution was prepared by dissolving 1 mg of each substance in 10 mL of HPLC-grade synthetic water. This stock solution was then added to the working solutions to obtain HA, FA, or NOM concentrations of 10 and 20 mg L^−1^. Experiments involving chloride addition were conducted by dissolving sodium chloride in the buffer solution immediately before the start of each experiment to achieve chloride concentrations of 50 and 100 mg L^−1^.

Throughout the experiments, the solutions were continuously stirred with a magnetic stirrer. After each step of electrooxidation, 0.1 mL of the treated solution was collected for LC-MS analysis and 1 mL was set aside for TOC measurement. Following each experiment, the electrodes were electrochemically cleaned in a sodium sulfate solution for 5 min at a constant current of 10 mA to remove adsorbed organic residues. The roles of the working and auxiliary electrodes were then reversed, and the cleaning process was repeated.

### 2.3. Analytical Procedures

The chromatography conditions were refined based on previous studies to ensure optimal performance. The LC–MS analysis was conducted using an Agilent high-resolution Q-TOF system series 6520 equipped with an electrospray ionization source (ESI). Separation was achieved using and Agilent 1290 Infinity UHPLC system, integrated with a G4212A diode array detector (DAD). The system was operated using MassHunter workstation software version B.06.00 (Agilent Technologies, Santa Clara, CA, USA), which facilitated data acquisition, as well as qualitative and quantitative analysis. A reference mass correction was implemented to ensure accurate mass measurements by utilizing lock masses of 121.050873 and 922.009798 in positive ion mode, and 112.98558700 and 1033.98810900 in negative ion mode. The optimization of instrument conditions began with tunning the MS detector in a positive or negative ionization mode, operating under an extended dynamic range of 2 GHz. The analysis was performed using a Zorbax RRHD Eclipse Plus-C18 (2.1 × 50 mm, dp = 1.8 μm) reversed phase chromatographic column (Agilent Technologies, Santa Clara, CA, USA). Detailed information regarding the chromatographic and spectrometric parameters are presented in Table S2.

The TOC concentration was measured with the TOC-L CPH analyser (Shimadzu Corporation, Kyoto, Japan), equipped with the high sensitivity platinum catalyst, and employed the NPOC (non-purgeable organic carbon) measurement method. System was controlled by the TOC-Control-L software (version 1.09).

All analyses were performed in duplicate. The analytical parameters used are summarized in Table S2.

### 2.4. Toxicity Assessment

Toxicity of cefuroxime axetil and its TPs was measured using five models provided by the Vega platform (version 1.1.5-b48, calculation core version 1.2.8): Fish Acute (LC50) Toxicity model (IRFMN) (version 1.0.0), Fish Chronic (NOEC) Toxicity model (IRFMN) (version 1.0.0), Fathead Minnow LC50 96h (EPA) (version 1.0.7), Algae Acute (EC50) Toxicity model (IRFMN) (version 1.0.0) and Algae Chronic (NOEC) Toxicity model (IRFMN) (version 1.0.0). The obtained results were then submitted to the principal component analysis (PCA) in order to facilitate interpretation and visualization of the multidimensional data. The chemometric analysis was performed using R 4.3.3 software (GNU Project).

## 3. Results and Discussion

### 3.1. Effect of Experimental Conditions on Cefuroxime Axetil Degradation

**Figure 1:**
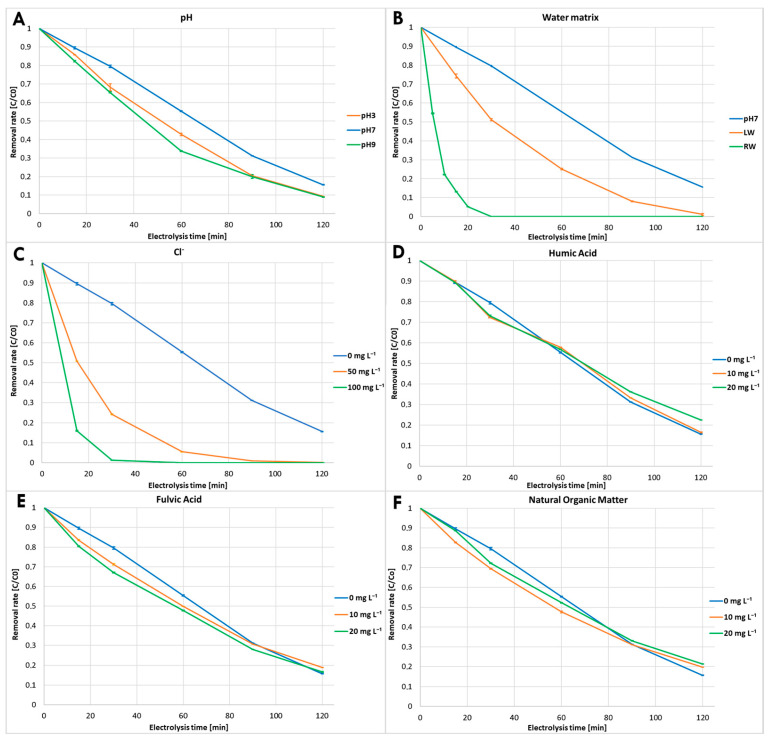
Kinetics of cefuroxime axetil electrochemical decomposition under different conditions ((**A**)—pH, (**B**)—natural lake and river water matrix, (**C**)—chloride concentrations, (**D**)—humic acid concentrations, (**E**)—fulvic acid concentrations, (**F**)—natural organic matter concentrations).

Extensive experimental trials revealed no decline in degradation efficiency attributable to electrode instability. Both electrodes demonstrated stable and reliable performance throughout the entire study period. To determine the degradation rate of cefuroxime axetil, the absorbance at 280 nm (UHPLC-DAD) was monitored throughout the electrolysis process. To assess the reaction kinetics, samples were collected from the reaction solution at 0, 15, 30, 60, 90, and 120 min of electrolysis for LC-MS and TOC (total organic carbon) analysis. In the case of the experiment conducted in river water, sampling was performed at 0, 5, 10, 15, 20, 30 min to capture the faster degradation dynamics observed in this matrix. The results clearly demonstrated that cefuroxime axetil undergoes significant electrooxidation, with the kinetics of the individual reactions presented in Table 1 and Figure 1.

#### 3.1.1. Effect of Initial pH Value

The initial pH plays a crucial role in the electrochemical oxidation process, which may affect the structure of organic matter, the generation and stability of reactive oxygen species, and the electrochemical behavior of coexisting ions [27]. Thus, in order to enhance the relevance of this study for potential practical applications, the influence of initial pH was determined over a wide pH range by adjusting the pH of the medium to 3.0, 7.0 and 9.0 before electrolysis. Different pH buffer values indicated a marked effect on the cefuroxime axetil degradation rate, as illustrated in Figure 1A. Both acidic and basic conditions resulted in more effective degradation of cefuroxime axetil neutral condition. Under basic conditions, degradation was initially more efficient than in acidic medium. However, after 90 min, the reaction rate slowed down and the final degradation efficiency became comparable to that observed under acidic conditions. By the end of the experiment, approximately 91% degradation was achieved in basic and acidic media, compared to 84% at pH 7. Degradation under neutral condition followed pseudo-zero order kinetic with t_1/2_ of 68.88 min, whereas degradation under acidic and basic conditions followed pseudo-first order with t_1/2_ of approximately 49.03 and 38.32 min, respectively.

#### 3.1.2. Degradation in Natural River and Lake Water Matrices

To assess the degradation rate of cefuroxime axetil in a natural water matrix containing a mixture of inorganic ions and organic matter, an experiment using both river and lake water was conducted. As shown in Figure 1B, lake water significantly enhanced the degradation of cefuroxime axetil, but the effect was even more pronounced in river water, where the substance degraded rapidly, reaching complete degradation in less than 30 min. In contrast, in lake water, approximately half of the substance was degraded within the same timeframe. The t_1/2_ for these reactions were 5.13 min for river water and 30.05 min for lake water, both following pseudo-first order kinetic.

River water, compared to lake water, was characterized by a higher concentration of inorganic ions, particularly chloride ions, and a lower TOC content, indicating reduced levels of organic matter. The presence of these components could have a significant influence on both the conductivity of the solution and the generation of reactive species, which mediated the electrochemical degradation of cefuroxime axetil. To further assess the influence of specific parameters on the degradation efficiency, additional experiments were conducted with the presence of DOM and with chloride addition.

#### 3.1.3. Effect of Chloride Addition

The role of mediators is crucial to examine due to their widespread presence in various types of wastewaters. Given the high concentration of chloride ions in river water, it is important to evaluate their role in the degradation of cefuroxime axetil to understand the reason behind the differences in effectiveness between river and lake water. High chloride concentrations are commonly found in many effluents. In solutions containing chloride ions, oxidation typically results in chlorine evolution. During electrochemical treatment, chloride ions may undergo oxidation on the BDD anode, forming oxychlorine species such as chlorate or perchlorate, which can be primarily attributed to the high activity of BDD anodes in generating •OH from water discharge [30].

To assess the impact of chloride ions on the degradation kinetics and mineralization efficiency of aqueous cefuroxime axetil solutions, experiments were conducted in a neutral medium (pH 7) with chloride concentrations of 50 and 100 mg L^−1^. The influence of chloride concentration on the electrochemical degradation of cefuroxime axetil is shown in Figure 1C. The degradation efficiency of cefuroxime axetil significantly increased with the addition of 50 mg L^−1^ of chloride, and further improved when the concentration was increased to 100 mg L^−1^, although this increase was less significant.

Nearly complete degradation of cefuroxime axetil was achieved within 120 min at a chloride concentration 50 mg L^−1^, and within 60 min at a concentration of 100 mg L^−1^. The results showed that the electrochemical degradation followed pseudo-first order kinetics for both chloride concentrations. At 100 mg L^−1^ of chloride, the degradation was more than 11 times faster compared to the process without chloride addition. These results can be explained by the increase in the conductivity of the solution and the enhanced degradation of pollutants through the involvement of active chlorine.

#### 3.1.4. Effect of Dissolved Organic Matter

DOM constitutes a vital component of natural organic matter in soil and water. Its effect on the electrochemical degradation of pollutants is complex. DOM can significantly enhance the solubility of relatively insoluble in water substances [31]. DOM, especially humic acid (HA) and fulvic acid (FA), plays a crucial role in enhancing the electrooxidation of pollutants by acting as electron shuttle mediators [32]. On the other hand, DOM can influence the oxygen levels in the solution, thereby influencing the course of redox reactions. Moreover, susceptibility of these substances to mineralization by anodic oxidation implies a potential to occupy active sites on the anode surface, thereby impeding reactions of the pollutants with reactive species.

The addition of various DOM fractions to the cefuroxime axetil solution had a noticeable effect on the reaction, although the differences were not significant. Interestingly, regardless of the fraction used—HA, FA, or NOM—the reaction profile remained similar. Similarly, increasing the concentration from 10 to 20 mg L^−1^ did not lead to significant changes in the reaction progression. In all experiments, the addition of DOM initially accelerated the reaction. However, the effect was more pronounced with FA and NOM compared to HA. Over time, the reaction slowed down, resulting in a poorer outcome after 2 h than in the experiment without any DOM addition. The fraction of cefuroxime axetil remaining in solution with 10 or 20 mg L^−1^ of each substance is presented in Figure 1D–F. The best results were obtained using 10 mg L^−1^ HA or 20 mg L^−1^ FA, achieving nearly 84% degradation, approaching the outcome observed in the solution without DOM addition.

### 3.2. Cefuroxime Axetil Mineralization

**Figure 2:**
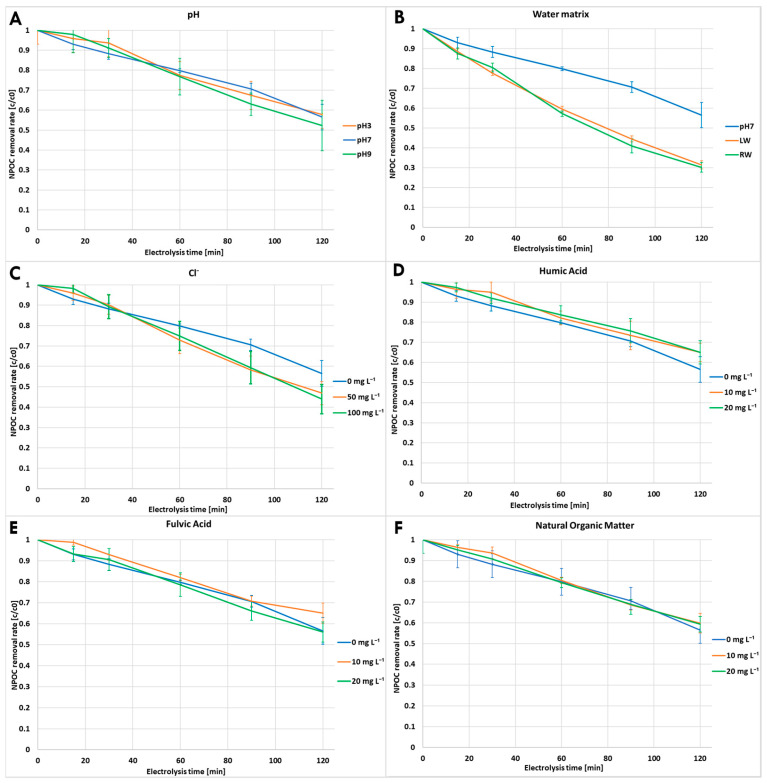
Cefuroxime axetil mineralization under different conditions ((**A**)—pH, (**B**)—natural lake and river water matrix, (**C**)—chloride concentrations, (**D**)—humic acid concentrations, (**E**)—fulvic acid concentrations, (**F**)—natural organic matter concentrations).

Considering that not only cefuroxime axetil, but also the resulting TPs, may exhibit significant pharmacological activity or be toxic to living organisms, complete mineralization is the most desirable outcome of the electrodegradation process. Degradation of complex organic molecules like cefuroxime axetil, involves multiple steps and may yield various intermediate products before complete mineralization to CO_2_, H_2_O, or nitrates and sulfates. Thus, it is reasonable to anticipate that the rate of TOC removal for a larger molecule like cefuroxime axetil, would be notably lower compared to the simple initial degradation of the compound. In this study, mineralization during the BDD-driven experiments was assessed by monitoring the reduction in NPOC concentration at 15, 30, 60, 90, and 120 min. The results are shown in Figure 2. As observed, even in experiments that rapidly led to the complete degradation of cefuroxime axetil (e.g., in the presence of chlorides or river water), the elimination of NPOC did not exceed 70%. In experiments with varying pH levels, it was observed that despite a higher degradation rate at pH 3 compared to pH 7, the reduction under neutral conditions was comparable, and even slightly better, than in the acidic environment. However, pH 9 proved to be the most effective for the mineralization of the tested substance.

Experiments conducted in river water or lake water significantly increased the mineralization rate compared to neutral conditions. However, the difference between the river water and lake water experiments was negligible. Despite the much faster degradation of cefuroxime axetil in river water than in lake water, the reduction of NPOC occurred in a very similar manner in both environments. The addition of chlorides resulted in similar outcomes; although a chloride concentration of 50 mg L^−1^ led to greater NPOC removal, increasing the concentration to 100 mg L^−1^ did not result in better NPOC removal. Moreover, while the addition of chlorides improved mineralization compared to neutral conditions, the NPOC reduction did not exceed 56%, which is significantly worse compared to experiments conducted in river water or lake water matrices. This observation suggests that the higher chloride content in the river water matrix, compared to lake water, may have contributed to greater degradation of cefuroxime axetil without simultaneously enhancing NPOC removal. The effect of different DOM on mineralization was quite similar. Generally, the addition of DOM to the solution decreased the mineralization rate. Only in the experiment with 20 mg L^−1^ of FA, did the NPOC reduction show a slight increase compared to the solution without DOM. Furthermore, it was found that this value deviated significantly from the experiment conducted at the lower concentration, where NPOC reduction, similar to the experiments with HA, was the least effective, reaching only 35% mineralization by the end of 2 h experiment. However, it should be noted that since total NPOC was measured, it is difficult to determine from the above results whether the decrease in cefuroxime mineralization was due to increased competition for reactive species with DOM, or if the components of the DOM themselves were less likely to undergo complete mineralization.

The aim of this study was both the identification of transformation products and the evaluation of the degradation efficiency of cefuroxime axetil using the BDD-driven process. It should be emphasized that although electrooxidation with BDD did not achieve complete mineralization, it demonstrated higher effectiveness in NPOC removal than many conventional water treatment methods [33,34]. As reported in numerous review studies, widely applied biological, adsorption-based, and coagulation processes typically lead only to partial transformation of organic pollutants or their transfer between phases, rather than to true mineralization. Even many advanced oxidation processes are often limited by the accumulation of persistent transformation products, which restricts their ability to fully remove organic carbon [35]. In this context, despite its limitations, BDD-driven treatment appears to be a promising approach for the treatment of waters contaminated with persistent pharmaceutical compounds.

At the same time, the practical applicability of electrochemical technologies is strongly dependent not only on their removal efficiency but also on their energy demand. Although higher current densities could further improve mineralization, they would also significantly increase energy consumption. For this reason, process efficiency was evaluated in terms of energy demand per gram of NPOC removal rather than by pursuing maximum mineralization. Electrochemical degradation of pollutants inherently requires energy, and the amount consumed is influenced by various operational parameters, including applied current, average cell voltage, reaction duration, and sample volume. Energy consumption (*E_c_*) was quantified in this study using Equation (1) [36].

(1) $$E_{c}=\frac{E_{cell}\times I\times t}{V_{s}\times ∆NPOC},$$

In this formula, *E_c_* represents the energy consumption, *E_cell_* is the average cell potential (V), *I* is the current (A), *t* is the reaction time (h), *V_s_* denotes the sample volume (L), and Δ*NPOC* reflects the reduction in non-purgeable organic carbon (in mg L^−1^). According to Equation (1), energy consumption increases linearly with the applied current, average cell voltage, and reaction time, reflecting the higher electrical input required under more intensive operating conditions. In contrast, higher sample volume and greater NPOC removal lead to lower specific energy consumption, as the supplied energy is distributed over a larger amount of degraded organic carbon. As presented in Table 2, energy requirements differed notably across experimental conditions, highlighting the sensitivity of the process to varying operational factors.

Comparison across different pH conditions revealed that more alkaline environments not only enhanced overall mineralization efficiency but also reduced energy consumption, indicating that alkaline pH conditions are more favorable for both the effectiveness and cost-efficiency of the electrochemical degradation process.

The addition of chlorides to the buffer resulted in an improvement in the energy efficiency of the process compared to the control. However, increasing the chloride loading did not lead to any further significant changes in energy efficiency. A substantial reduction in energy consumption was observed across all experiments involving DOM addition, with higher organic matter loading further decreasing energy demand in each case. The impact of DOM was so pronounced that experiments with HA, FA, and NOM at a loading of 20 mg L^−1^ resulted in lower energy consumption than any changes induced by pH adjustment or chloride addition. However, despite the improved process efficiency at elevated DOM concentrations, the specific fraction of organic carbon responsible for this effect remains unclear (similar as in the case of mineralization rate).

Finally, experiments conducted in natural matrices—river water and lake water—demonstrated that the presence of a complex organic matrix can lead to lower energy consumption per gram of organic matter. However, it is difficult to fully interpret this effect, as these matrices contain a mixture of inorganic ions and natural organic matter. Inorganic ions may increase conductivity and potentially enhance degradation through indirect oxidation mechanisms (e.g., formation of secondary reactive species), whereas the organic matrix could limit radical interactions with cefuroxime axetil. Nevertheless, the observed low energy consumption per gram of organic matter, achieved despite the relatively high cell voltages applied, suggests that the rapid degradation of other organic compounds present in the solution played a significant role.

### 3.3. Degradation Intermediates

Incomplete mineralization of the solution, despite a high degradation efficiency of the target compound, may result from the formation of intermediate by-products or the accumulation of low-molecular-weight organic acids. Such partial oxidation pathways are common in advanced oxidation and electrochemical processes and do not necessarily indicate an unfavorable treatment outcome. The transformation of complex pollutants into short-chain organic acids and other low-molecular-weight oxygenated intermediates, is generally considered environmentally acceptable, as these compounds are less persistent and often more biodegradable [37].

Identification of TPs is essential when evaluating degradation methods for wastewater treatment, as degradation does not always mean detoxification. This is because degradation of a parent compound does not necessarily equate to its detoxification. In many cases, the TPs formed during the treatment process can be more toxic, persistent, or bioaccumulative than the original substance. Their identification helps assess environmental safety, prevents the formation of harmful byproducts, and provides insight into degradation pathways and process efficiency. In total, ten electrooxidation products of cefuroxime axetil were identified during the experiments, and their molecular structures were characterized using high-resolution mass spectrometry (MS) analysis. The structures and fragmentation pattern of cefuroxime axetil and its TPs are summarized in Table 3.

For more accurate identification of certain compounds, analysis in negative ionization mode was also employed, with the resulting fragmentation patterns presented in Table S3. The MS/MS spectra, showing the fragmentation ions of the formed TPs, along with a discussion on their identification, are presented in the Supplementary Materials (Section S1, Figures S2–S19). Two diastereoisomers of cefuroxime axetil are separated by the developed chromatographic method. Electrochemical TPs, which retained this stereogenic center, were also visible on the chromatograms as two peaks. The main TPs are two monooxygenated products. In the case of TP1, hydroxylation occurred in the position 6 of ß-lactam ring, while TP2 is sulfoxide. Both products were particularly formed in natural waters and chloride-rich environments, where they represented the main products, whereas the presence of DOM had a negative effect on their formation efficiency. An alkaline environment did not favor the formation of either of these oxidation products. At pH 9, TP1 was not formed, and TP2 was generated only in trace amounts.

Oxidation under neutral and alkaline conditions led to the formation of TP3, in which experiments it was the main degradation product. TP3 resulted from a double oxidation of the ß-lactam ring, and two hydroxyl groups are situated at positions 2 and 3, where the double bond was originally located. This product was also generated in significant amounts in experiments with the addition of DOM, whereas the presence of chlorides significantly inhibited its formation. In experiments with chlorides and in river water matrix, the product was formed only in trace amounts; interestingly, however, the lake water matrix yielded opposite results, highlighting the crucial role of chemical composition of the water matrix in determining the direction of oxidation processes.

TP4 (2-(furan-2-yl)-2-(methoxyimino)acetamide) is a degradation product that resulted from the cleavage of the β-lactam ring. The formation of this product was pH-dependent; it was most efficiently formed under alkaline conditions, while no formation was observed at pH 3. In the presence of DOM, it was produced in small amounts. A significant increase in its formation was observed in the presence of chloride ions. Additionally, its formation was notably higher in the river water matrix compared to lake water.

TP5 and TP6 are chlorinated organic products resulting from mono-oxidation and the addition of a single chlorine atom within the ß-lactam structure. In the case of TP5, the chlorine atom is attached to position 2, while the hydroxyl group is located at position 6. TP 6 is sulfoxide, with the chlorine atom most likely attached to position 6 of the ß-lactam ring. Both products were formed in the experiment with added chlorides, whereas in the river water matrix only TP5 was detected. In the lake water matrix, neither of these products was formed, despite the presence of chlorides in this matrix.

The next product, TP7—anti-cefuroxime axetil diastereoisomers (A and B epimers)—was formed only in the experiment conducted at pH 3. Since literature data indicate that isomerization to these two epimers occurs under acidic conditions, a control experiment was carried out, which confirmed that the formation of this product should be attributed to electrochemical processes.

TP8 and TP9 are additional products formed exclusively under pH 3 conditions. They are isomers of the oxidation products TP1 and TP2, derived from the oxidation of anti-configurational cefuroxime axetil diastereoisomers.

The last detected product, TP10, is an axetil-free cefuroxime acid. Formation of this product was only observed in alkaline conditions.

**Figure 3:**
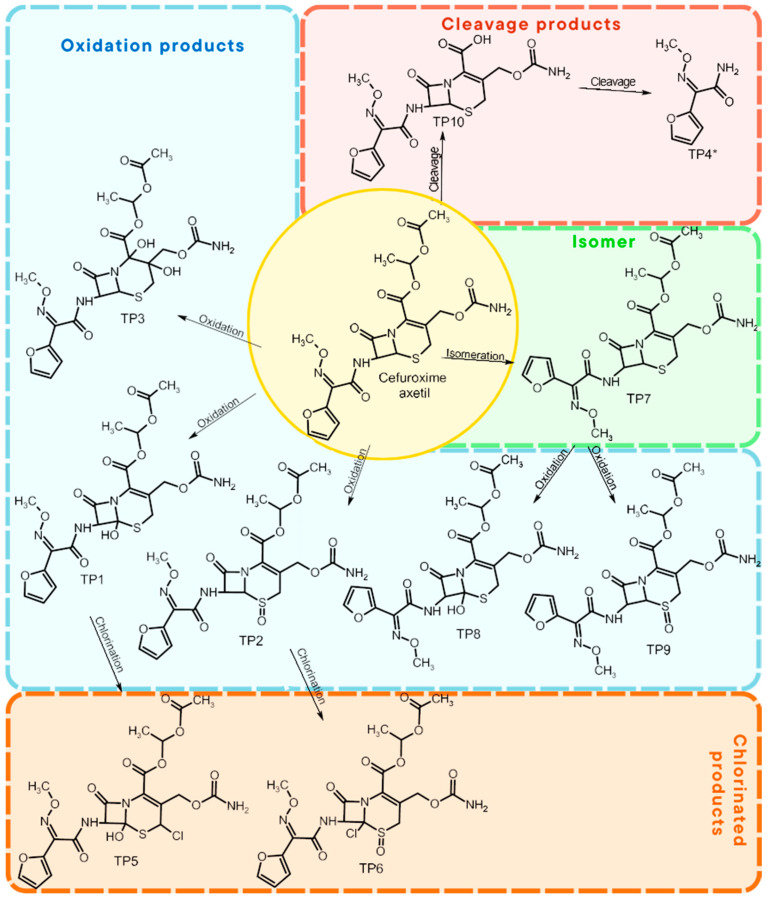
Proposed transformation pathway of cefuroxime axetil during electrochemical experiments (* TP4, identified as a cleavage product, may originate not only from the parent cefuroxime molecule but also from its degradation products).

Four known transformation products of cefuroxime axetil (TP2, TP7, TP9, and TP10), previously reported in the literature under various stress conditions, were also observed in our electrochemical degradation experiments [3,38,39]. To the best of our knowledge, the remaining six products have not been previously reported. These include the oxidation product TP1 and its structural isomer TP8, the double-oxidation product TP3, the degradation product TP4, as well as the chloro-oxidized derivatives TP5 and TP6. The proposed transformation pathway is presented in Figure 3.

### 3.4. Aquatic Toxicity of the TPs

**Figure 4:**
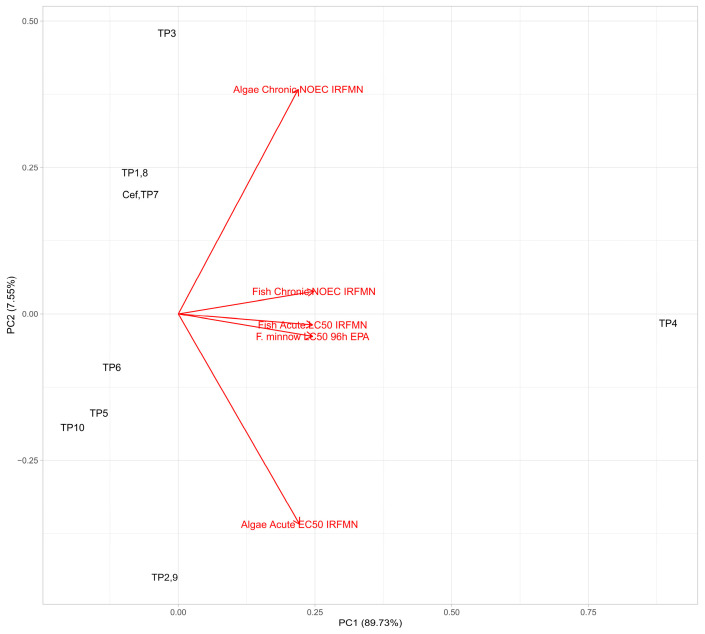
Plot of principal component analysis of calculated toxicity of cefuroxime axetil TPs toward aquatic organisms.

Particular attention must be paid to intermediate species exhibiting newly formed or previously uncharacterized molecular structures, as their toxicological profiles cannot be assumed a priori. Therefore, in this study we evaluated acute and chronic toxicity of the identified intermediates using five in silico models: three concerning fish (including one designed specifically for *F. minnow*) and two concerning algae. In order to facilitate interpretation and visualization of the calculated toxicity values, PCA was performed. As shown in Figure 4 the models predicting toxicity towards fish gave rather correlated results (vectors close to parallel). The most outlying (and at the same time the least toxic to fish) compound is TP4, which matches the raw data shown in the Supplementary Material (Table S4). Among the remaining TPs relatively low toxic to fish were TP2 and TP9 (sulfoxides). On the other hand, TP10 (cefuroxime with the detached axetil moiety) turned out to be comparatively harmful to fish (but excluding the *F. minnow* model). The models predicting toxicity towards algae gave differing results, dividing the detected products into two groups: compounds possessing relatively low acute and high chronic toxicity—TP5, TP6 (chloro-oxidized), TP2, TP9 (sulfoxides) and TP10 (cefuroxime, exceptionally possessing comparatively high chronic and acute toxicity). The second group of compounds consists of products possessing relatively low chronic and high acute toxicity to algae: parent compound along with TP7 (its structural isomer), TP1 with TP8 (hydroxylated derivatives) and TP3 (doubly hydroxylated and hydrogenated product).

On the basis of the obtained data, it can be stated that in case of the studied drug the sulfoxidation decreases toxicity to fish and acute toxicity to algae (but increases chronic algal toxicity). Chlorination combined with oxidation lowers acute and elevates chronic toxicity to algae. Opposite relationship is observed in the case of hydroxylation and double hydroxylation. Detachment of the axetil moiety results in overall higher aquatic toxicity according to the applied models. Generally, it should be noted that the differences in toxicity between the parent compound and its TPs are rather modest. The only exception is TP4, possessing considerably lower toxicity, probably due to significantly higher polarity and lower molecular mass.

## 4. Conclusions

In the present study, the influence of various conditions on the removal efficiency of cefuroxime axetil was investigated using an electrochemical oxidation process with a BDD electrode. The conducted studies revealed that cefuroxime axetil is susceptible to electrooxidation.

The pH of the reaction played a significant role, with the degradation being more efficient under basic and alkaline conditions compared to neutral pH. Experiments conducted in lake water and river water showed substantial improvement in degradation rate, particularly in river water, where the t_1/2_ was reduced to 5.13 min. To better understand the differences between these matrices, additional experiments were carried out to investigate the influence of DOM and chlorides on degradation efficiency. The addition of DOM caused a slight decrease in reaction efficiency, whereas the presence of chlorides led to a marked increase in degradation. This effect was further enhanced when the chloride concentration was increased to 100 mg L^−1^, under which conditions the degradation t_1/2_ reached 5.95 min.

In total, ten electrooxidation products of cefuroxime axetil were identified, and their structures were elucidated using high-resolution MS analysis. As a result of oxidation, three main products were formed: a hydroxylated compound, a sulfoxide, and a dihydroxylated compound resulting from saturation of a double bond through the addition of two hydroxyl groups. Two cleavage products were formed to a lesser extent, indicating partial breakdown of the parent compound. In the presence of chlorine, chlorinated organic products were detected, although in low concentrations. While their formation indicates some degree of chlorination during treatment, it does not significantly compromise the overall safety of the method when properly controlled. Additionally, the formation of an isomer was observed, which was further oxidized into two additional oxidation TPs.

The TOC analysis proved to be crucial for accurately evaluating the efficiency of the process, as the results indicate that the rate of compound degradation does not always directly correlate with the extent of mineralization. The highest NPOC elimination values were observed in experiments conducted in river water and lake water matrices, where NPOC removal reached nearly 70%. Mineralization in lake water proceeded at a similar rate to that in river water, despite the slower degradation of the compound. In contrast, mineralization in the presence of chlorides was less efficient, even though the degradation of the compound occurred just as rapidly as in the river water matrix. Mineralization efficiency increased with rising buffer pH. Additionally, higher concentrations of DOM and the presence of chlorides were associated with a more energy-efficient process.

The performed in silico predictions showed that the TPs formed as a result of the studied electrochemical processes do not differ significantly from the parent molecule in the term of aquatic toxicity, and the only outlying compound is the significantly less harmful product of cefuroxime axetil breakdown. These findings clearly shows that high mineralization level of the studied drug is a highly desirable outcome. However, on the other hand, lack of substantially more toxic intermediates indicates that the explored degradation process does not increase the environmental risk, even in the presence of chlorides.

The demonstrated efficiency under variable pH and in natural water matrices highlights the potential of electrochemical-based AOP with BDD electrodes for real-world applications, offering rapid, effective, cost-efficient, and environmentally friendly performance. While this approach is highly effective at degrading pollutants and breaking them down into smaller intermediate compounds, complete mineralization may not always be achieved. Therefore, its effectiveness and the minimization of residual by-products could be further enhanced by combining it with complementary treatment techniques in wastewater treatment plants, ensuring a more thorough removal of contaminants.

## Figures and Tables

**Table 1:** Summary of the electrolysis kinetics.

Matrix	Loading	Model	Fit (r)	k [min^−1^]	t_1/2_ [min]
pH3	-	Pseudo-first order	0.9899	0.01797	49.03
pH7	-	Pseudo-zero order	0.9980	0.0073 *	68.88
pH9	-	Pseudo-first order	0.9958	0.0200	38.32
River Water	-	Pseudo-first order	0.9967	0.1350	5.13
Lake Water	-	Pseudo-first order	0.9743	0.0231	30.05
Chlorine	50 mg L^−1^	Pseudo-first order	0.9981	0.0485	14.29
Chlorine	100 mg L^−1^	Pseudo-first order	0.9940	0.1164	5.95
Humic Acid	10 mg L^−1^	Pseudo-zero order	0.9960	0.0070 *	71.69
Humic Acid	20 mg L^−1^	Pseudo-zero order	0.9942	0.0065 *	76.95
Fulvic Acid	10 mg L^−1^	Pseudo-first order	0.9964	0.0116	59.83
Fulvic Acid	20 mg L^−1^	Pseudo-first order	0.9958	0.0123	56.23
NOM	10 mg L^−1^	Pseudo-first order	0.9990	0.0123	56.14
NOM	20 mg L^−1^	Pseudo-first order	0.9970	0.0108	64.09

* For zero-order reactions, k is expressed in mg L^−1^ min^−1^.

**Table 2:** Influence of various factors on energy consumption in the electrochemical mineralization of cefuroxime axetil (in kWh per g of NPOC).

Matrix	Loading	Energy Consumption [kWh g^−1^]
pH3	-	2.20
pH7	-	1.96
pH9	-	1.67
River Water	-	1.36
Lake Water	-	0.60
Chlorine	50 mg L^−1^	1.61
Chlorine	100 mg L^−1^	1.61
Humic Acid	10 mg L^−1^	1.67
Humic Acid	20 mg L^−1^	1.18
Fulvic Acid	10 mg L^−1^	1.57
Fulvic Acid	20 mg L^−1^	0.92
NOM	10 mg L^−1^	1.48
NOM	20 mg L^−1^	1.02

**Table 3:** Accurate masses and proposed structures of the TPs of cefuroxime axetil formed during electrolysis.

Name	Structure	t_R_	Mass [m/z]		Error [ppm]	Elemental Formula *	Fragmentation (MS/MS)		Reaction Conditions						
			Theoret.	Experim.			Mass [m/z]	Elemental Formula	pH3	pH7	pH9	RW	LW	DOM	Cl^−^
Cef axet	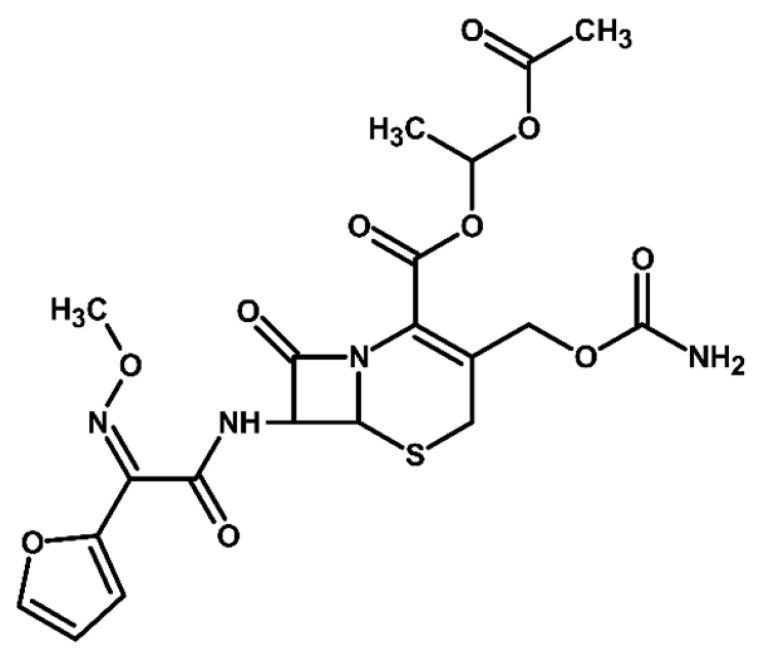	5.9 6.05	533.0949	533.0958	1.69	C_20_H_22_N_4_NaO_10_S	489.0684 447.0581 403.0664 386.0422 359.0795 342.0519 314.0580 298.0625 275.0091 231.0382 175.0069 134.0219	C_18_H_18_N_4_NaO_9_S C_16_H_16_N_4_NaO_8_S C_15_H_16_N_4_NaO_6_S C_15_H_13_N_3_NaO_6_S C_13_H_17_N_3_NaO_7_S C_14_H_13_N_3_NaO_4_S C_13_H_13_N_3_NaO_3_S C_12_H_14_N_2_O_5_S C_10_H_8_N_2_NaO_4_S C_9_H_8_N_2_NaO_4_ C_6_H_4_N_2_NaOS C_4_H_8_NO_2_S							
TP1	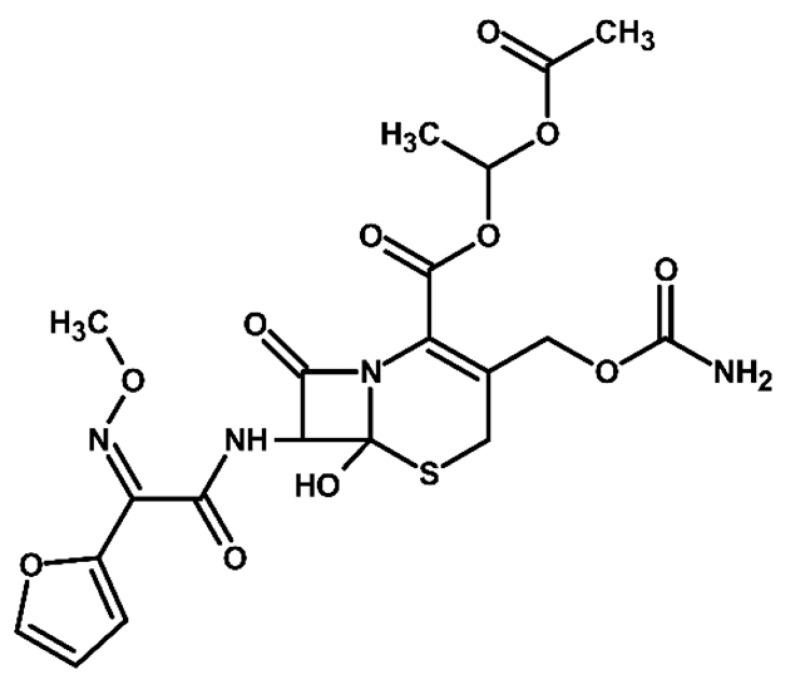	4.8 4.9	549.0898	549.0892	1.09	C_20_H_22_N_4_NaO_11_S	505.0575 463.0513 445.0389 419.0594 402.0358 375.0662 358.0440 340.0304 330.0515 291.0053 265.0259 231.0348 222.0237 203.0244 161.0078 151.0480 134.0245 84.0054	C_18_H_18_N_4_NaO_10_S C_16_H_16_N_4_NaO_9_S C_16_H_14_N_4_NaO_8_S C_15_H_16_N_4_NaO_7_S C_15_H_13_N_3_NaO_7_S C_13_H_17_N_3_O_8_S C_14_H_13_N_3_NaO_5_S C_14_H_10_N_3_NaO_4_S C_13_H_13_N_3_NaO_4_S C_10_H_8_N_2_NaO_5_S C_9_H_10_N_2_NaO_4_S C_9_H_8_N_2_NaO_4_ C_8_H_9_NNaO_3_S C_7_H_9_NO_4_S C_5_H_7_NO_3_S C_5_H_8_N_2_NaO_2_ C_4_H_8_NO_2_S C_3_H_2_NO_2_	+	+	-	+	+	+	+
TP2	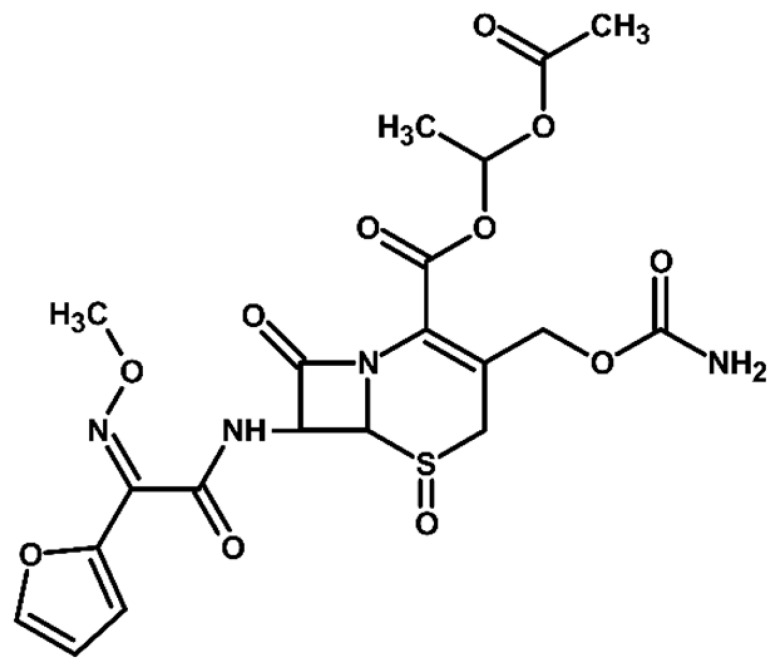	5.1 5.2	549.0898	549.0889	1.64	C_20_H_22_N_4_NaO_11_S	463.0558 419.0669 402.0377 358.0475 291.0056 265.0261 231.0401 222.0266 195.0355 151.0496 134.0207	C_16_H_16_N_4_NaO_9_S C_15_H_16_N_4_NaO_7_S C_15_H_13_N_3_NaO_7_S C_14_H_13_N_3_NaO_5_S C_10_H_8_N_2_NaO_5_S C_9_H_10_N_2_NaO_4_S C_9_H_8_N_2_NaO_4_ C_7_H_9_N_3_NaO_2_S C_8_H_7_N_2_O_4_ C_5_H_8_N_2_NaO_2_ C_4_H_8_NO_2_S	+	+	+	+	+	+	+
TP3	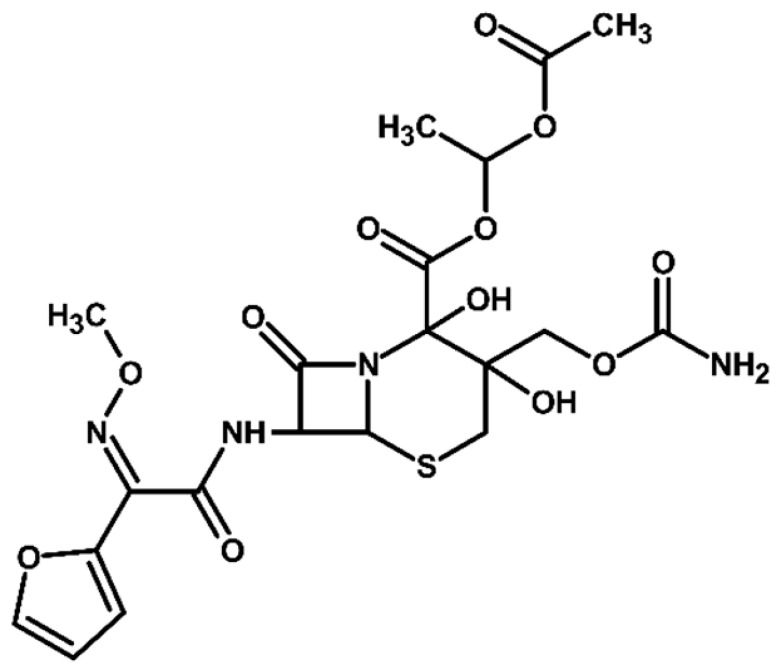	3.6	567.1004	567.0994	1.76	C_20_H_24_N_4_NaO_12_S	523.0733 481.0632 437.0739 393.0851 376.0556 358.0435 332.0600 283.0334 265.0422 133.0356	C_18_H_20_N_4_NaO_11_S C_16_H_18_N_4_NaO_10_S C_15_H_18_N_4_NaO_8_S C_13_H_19_N_3_NaO_9_S C_14_H_15_N_3_NaO_6_S C_14_H_13_N_3_NaO_5_S C_13_H_15_N_3_NaO_4_S C_9_H_12_N_2_NaO_5_S C_9_H_10_N_2_NaO_4_S C_4_H_9_N_2_OS	+	+	+	+	+	+	+
TP4	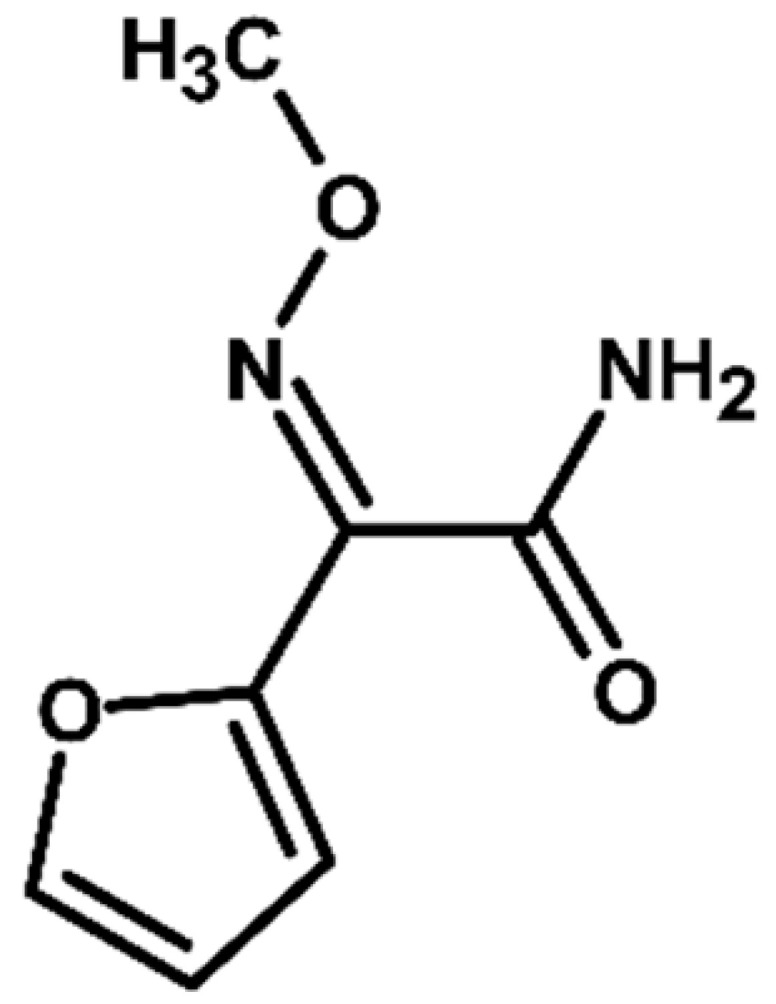	1.8	191.0427	191.0434	3.66	C_7_H_8_N_2_NaO_3_	-	-	-	+	+	+	+	+	+
TP5	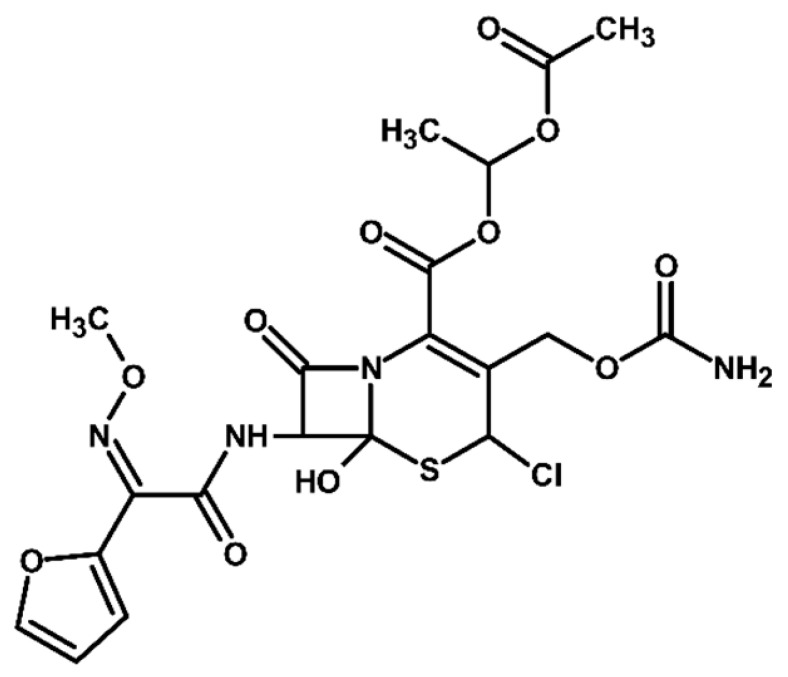	6.2	583.0508	583.0519	1.89	C_20_H_21_ClN_4_NaO_11_S	539.0251 497.0156 417.0433 400.0155 374.0359 356.0283 291.0077 231.0307 200.0202 185.0065	C_18_H_17_ClN_4_NaO_10_S C_16_H_15_ClN_4_NaO_9_S C_15_H_14_N_4_NaO_7_S C_15_H_11_N_3_NaO_7_S C_14_H_13_N_3_NaO_6_S C_14_H_11_N_3_NaO_5_S C_10_H_8_N_2_NaO_5_S C_9_H_8_N_2_NaO_4_ C_7_H_6_NO_6_ C_5_H_7_ClN_2_NaO_2_	-	-	-	+	-	-	+
TP6	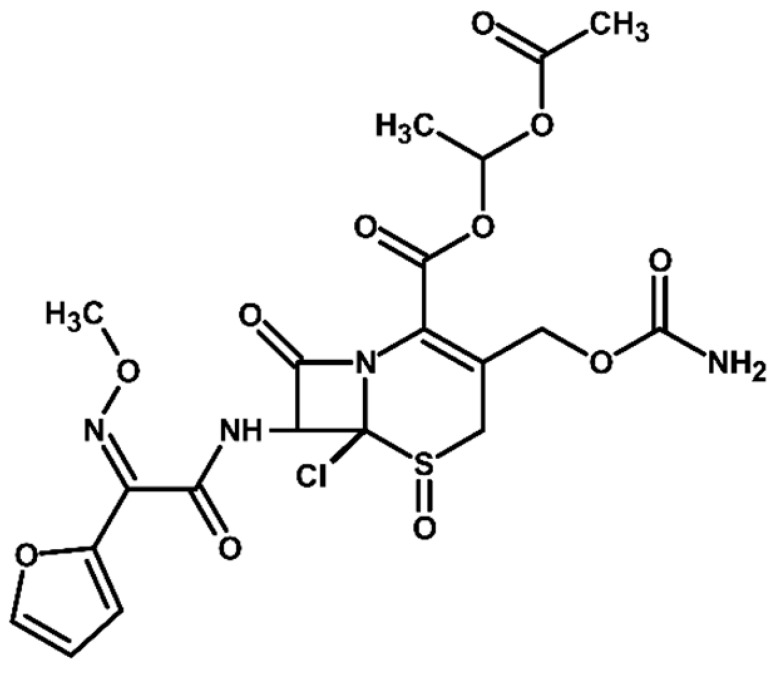	5.5	583.0508	583.0514	1.03	C_20_H_21_ClN_4_NaO_11_S	547.0783 539.0224 497.0162 400.0205 291.0072 255.9874 182.9930	C_20_H_20_N_4_NaO_11_S C_18_H_17_ClN_4_NaO_10_S C_16_H_15_ClN_4_NaO_9_S C_15_H_11_N_3_NaO_7_S C_10_H_8_N_2_NaO_5_S C_7_H_8_ClN_3_NaO_2_S C_5_H_5_ClN_2_NaO_2_	-	-	-	-	-	-	+
TP7	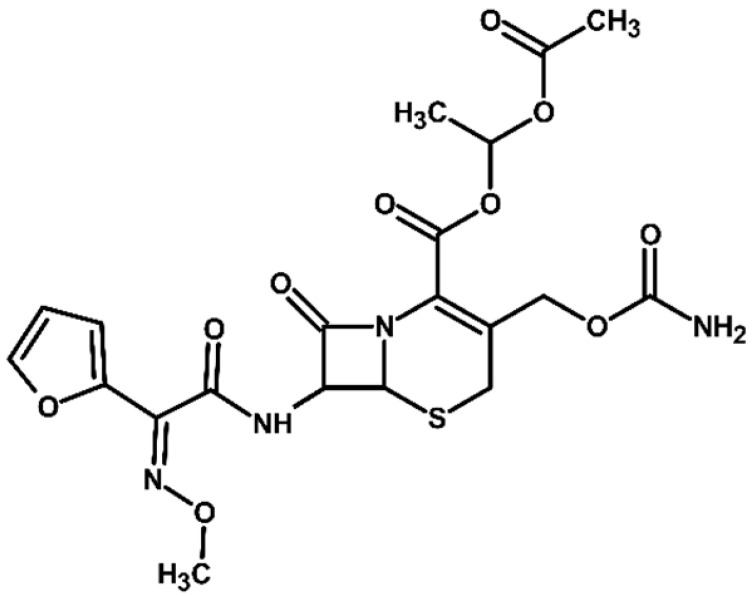	6.51 6.65	533.0949	533.0937	2.25	C_20_H_22_N_4_NaO_10_S	489.0647 447.0563 386.0420 359.0769 342.0490 314.0561	C_18_H_18_N_4_NaO_9_S C_16_H_16_N_4_NaO_8_S C_15_H_13_N_3_NaO_6_S C_13_H_17_N_3_NaO_7_S C_14_H_13_N_3_NaO_4_S C_13_H_13_N_3_NaO_3_S	+	-	-	-	-	-	-
TP8	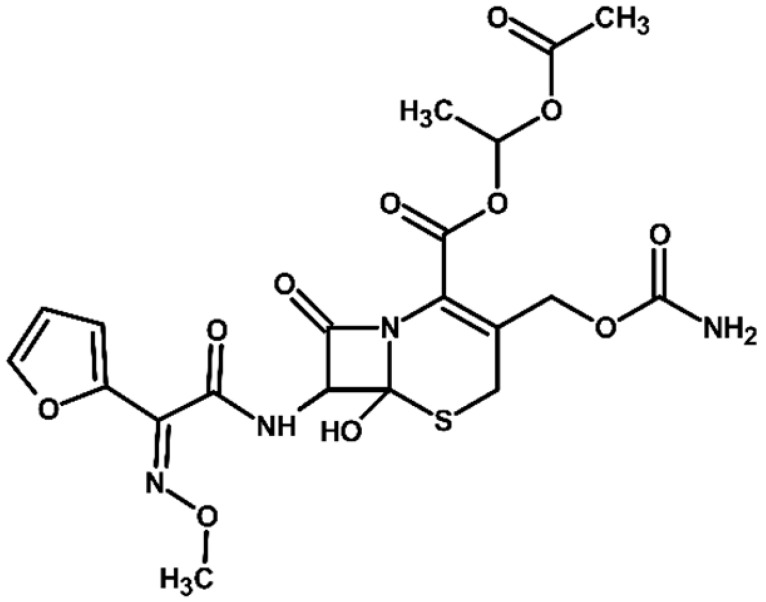	5.38 5.58	549.0898	549.0890	1.46	C_20_H_22_N_4_NaO_11_S	463.0567 402.0346 358.0488 330.0516	C_16_H_16_N_4_NaO_9_S C_15_H_13_N_3_NaO_7_S C_14_H_13_N_3_NaO_5_S C_13_H_13_N_3_NaO_4_S	+	-	-	-	-	-	-
TP9	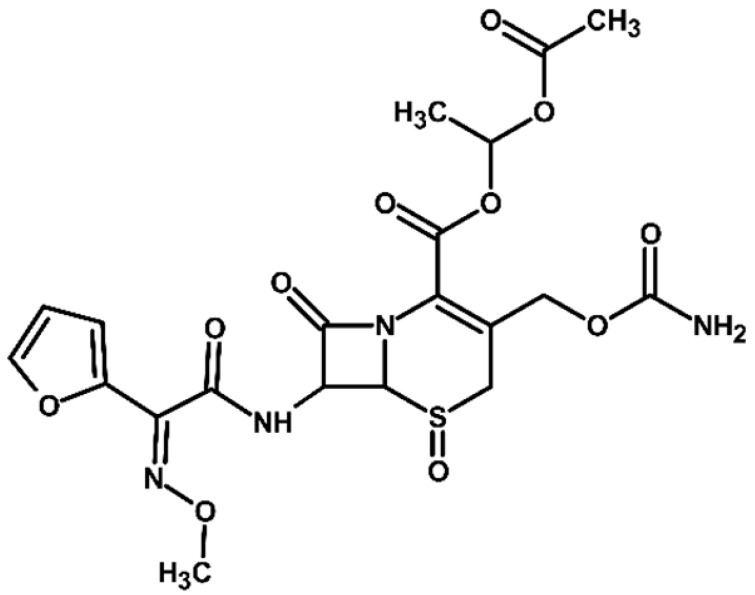	5.69 5.84	549.0898	549.0906	1.46	C_20_H_22_N_4_NaO_11_S	463.0503 402.0360 358.0244	C_16_H_16_N_4_NaO_9_S C_15_H_13_N_3_NaO_7_S C_14_H_13_N_3_NaO_5_S	+	-	-	-	-	-	-
TP10	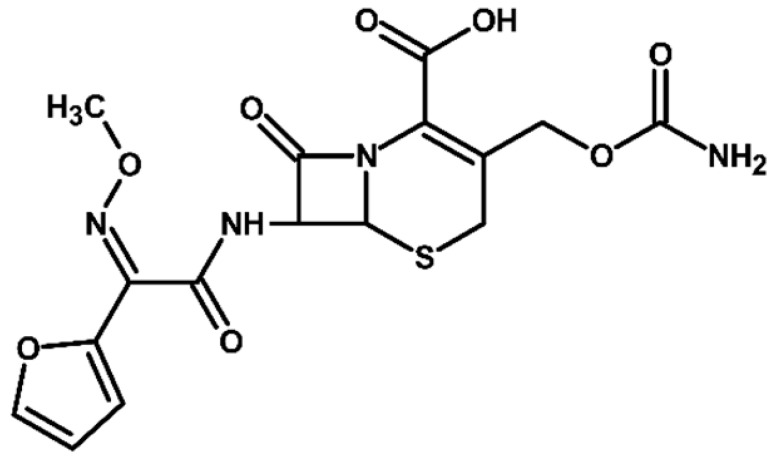	3.5	447.0578	447.0599	4.69	C_16_H_16_N_4_NaO_8_S	403.0606 386.0416 231.0349 175.0009	C_15_H_16_N_4_NaO_6_S C_15_H_13_N_3_NaO_6_S C_9_H_8_N_2_NaO_4_ C_6_H_4_N_2_NaOS	-	-	+	-	-	-	-

* All elemental formulas and MS masses are presented as sodium adducts. “+”—Observed. “-“—Not observed.

## Data Availability

Data are contained within this article or the Supplementary Materials.

## Supplementary Materials

The following supporting information can be downloaded at: https://www.mdpi.com/article/10.3390/molecules31010106/s1, Figure S1: The structures of diastereoisomers A and B of cefuroxime axetil, with retention times of 6.05 and 5.90 min, respectively, using the applied method, Figure S2: MS/MS spectrum and fragmentation pattern of cefuroxime axetil in positive ion mode, Figure S3: MS/MS spectrum and fragmentation pattern of cefuroxime axetil in negative ion mode, Figure S4: MS/MS spectrum and fragmentation pattern of TP1 in positive ion mode, Figure S5: MS/MS spectrum and fragmentation pattern of TP1 in negative ion mode, Figure S6: MS/MS spectrum and fragmentation pattern of TP2 in positive ion mode, Figure S7: MS/MS spectrum and fragmentation pattern of TP2 in negative ion mode, Figure S8: MS/MS spectrum and fragmentation pattern of TP3 in positive ion mode, Figure S9: MS/MS spectrum and fragmentation pattern of TP3 in negative ion mode, Figure S10: MS/MS spectrum and fragmentation pattern of TP5 in positive ion mode (part 1), Figure S11: MS/MS spectrum and fragmentation pattern of TP5 in positive ion mode (part 2), Figure S12: MS/MS spectrum and fragmentation pattern of TP5 in positive ion mode (part 3), Figure S13: MS/MS spectrum and fragmentation pattern of TP6 in positive ion mode (part 1), Figure S14: MS/MS spectrum and fragmentation pattern of TP6 in positive ion mode (part 2), Figure S15: MS/MS spectrum and fragmentation pattern of TP6 in positive ion mode (part 3), Figure S16: MS/MS spectrum and fragmentation pattern of TP7 in positive ion mode, Figure S17: MS/MS spectrum and fragmentation pattern of TP8 in positive ion mode, Figure S18: MS/MS spectrum and fragmentation pattern of TP9 in positive ion mode, Figure S19: MS/MS spectrum and fragmentation pattern of TP10 in positive ion mode; Table S1: Parameters of River water and Lake water, Table S2: LC-MS and TOC parameters, Table S3: Accurate masses of the TPs of cefuroxime axetil formed during electrolysis in negative mode, Table S4: NPOC data in conducted experiments, Table S5: Aquatic toxicity of cefuroxime axetil and its TPs [mg L^−1^].

## Abbreviations

The following abbreviations are used in this manuscript:

BDD	Boron-doped diamond
PBPs	Penicillin-binding proteins
FA	Fulvic acid
HA	Humic acid
TOC	Total organic carbon
TP	Transformation Product
