# Supplementary material for: Electrochemical Removal of Cephalosporin Antibiotic—Cefuroxime Axetil from Aquatic Media Using Boron-Doped Diamond Electrodes: Process Optimization, Degradation Studies and Transformation Products Characterization
Source: Molecules. 2025 Dec 26;31(1):106. doi: 10.3390/molecules31010106 (PMC12786646; doi:10.3390/molecules31010106)
Supplement: Supplementary file 1 [file molecules-31-00106-s001.zip › molecules-4029660-supplementary.pdf]

Supplementary material

Electrochemical Removal of Cephalosporin Antibiotic—Cefuroxime Axetil from Aquatic Media Using Boron-Doped Diamond Electrodes: Process Optimization, Degradation Studies and Transformation Products Characterization

Michał Wroński, Jakub Trawiński and Robert Skibiński *

Department of Medicinal Chemistry, Faculty of Pharmacy, Medical University of Lublin, Jaczewskiego 4, 20-090 Lublin, Poland

*Correspondence: robert.skibinski@umlub.pl; Tel.: +48-81-4487383

Section S1.

In positive mode, as a result of the presence of sodium ions in the mobile phase, sodium adducts were formed during electrospray ionization. In the MS/MS spectrum in positive mode of cefuroxime axetil, presented in Figure S2. Fragmentation begins with the removal of the axetil group. First, the acetyl moiety is lost, resulting in a peak at 489.0684 m/z, followed by the loss of the remaining fragment, leading to a peak at m/z 447.0581 corresponding to cefuroxime. A small peak 403.0664 m/z represents loss of single oxygen atom from carboxyl group. A prominent peak 386.0422 m/z resulted from detachment of acetamide with the cyclization to well-known lactone. Further loss of carbon dioxide results in a peak with 342.0519 m/z. Further fragmentation leads to loss of the methoxy group to form structure at 314.0580 m/z. Alternatively, a degradation of ß-lactam ring can occur, resulting in a peak with 275.0091 m/z, where after removal of nitrogen and three carbon atoms a cyclization to 2H-thiet-2-one. Next peak with m/z of 231.0382 represents molecule after the loss of sulfur and carbon atoms. Another peak, representing a degraded ß-lactam ring with removed sulfur atom is represented by the peak with m/z of 134.0219. A peak at 175.0069 m/z arises from the decarbonylation at position 8 of the β-lactam ring followed by intramolecular cyclization. Fragmentation from the other side leads removal of (furan-2-yl)(methoxyimino)acetaldehyde represented by a fragment with a m/z of 359.0795.

In the MS/MS spectrum in negative ion mode (Figure S3) the first peak at 466.0904 m/z corresponds to descarbamoyl cefuroxime axetil. A small peak at 60.0109 m/z represents acetamide, formed by cleavage from the fragment at 466.0904 m/z. Following the degradation of the axetil moiety, the resulting fragment forms a lactone at m/z 362.0459 m/z, which subsequently loses carbon dioxide to yield a peak at 318.0557 m/z. An alternative pathway, involving partial removal of the axetil group and loss of a hydroxymethyl group, leads to a fragment at 378.0753 m/z. A significant peak at 405.0503 m/z represents a fragment formed after complete removal of the axetil moiety, loss of an oxygen atom, and cyclization. Further fragmentation leads to partial degradation of ß-lactam ring, producing a peak at 284.0700 m/z, which, after the loss of methylamine and cyclization, yields a peak at 251.0152 m/z. Subsequent removal of carbon and sulfur atoms forms a peak at 207.0412 m/z is formed, which is the most intense signal in this MS/MS spectrum. Following the loss of oxygen a peak at 176.0143 m/z is formed. Peaks at 92.0143 and 116.0350 m/z are attributed to the cleavage of the ß-lactam ring.

The MS/MS spectrum (Figure S4) of TP1 shares many common fragments with the parent, differing in mass, indicating the presence of a hydroxyl group. Fragmentation begins with the removal of the axetil group. In positive ion mode, the first peak at m/z of 505.0659 results from the loss of acetyl moiety, followed by the loss of the remaining fragment, leading to a peak the most prominent peak in this spectrum at m/z 463.0513, which corresponds to cefuroxime with additional hydroxyl group. A small peak at 445.0389 m/z represents cefuroxime after loss of an attached oxygen atom as water. Alternatively, a descarboxycefuroxime retaining the hydroxyl group can be formed, observed at 419.0594 m/z. A lactone structure is also formed, represented by a significant peak at 402.0358 m/z. This lactone, after the loss of carbon dioxide, yields a peak at 358.0440 m/z, followed by the loss of a hydroxyl group, resulting in a fragment at 340.0304 m/z. Degradation of the ß-lactam ring leads to a formation of a peak at 291.0041 m/z, where the ß-lactam ring is transformed into a 2H-thiet-2,4-dione structure. This fragment corresponds to the 275.0091 m/z fragment observed in the parent MS/MS spectrum, but with an additional hydroxyl group. This is direct evidence, that the location of this additional hydroxyl group within ß-lactam ring can be only at position 1, 6 or 7. The peak at 265.0261 m/z corresponds to an intact ß-lactam ring with a hydroxyl group. The peaks at 203.0244 and 161.0078 m/z indicate partial degradation of the ß-lactam ring, with the attached hydroxyl group retained in the resulting fragments. These fragments exclude placement of hydroxyl group at position 7, supporting the hypothesis that the hydroxyl group is located at position 6. Another fragment representing a partially degraded ß-lactam ring is observed at m/z value of 134.0178. Following the elimination of the methylthio substituent, it is converted into a fragment at 84.0054 m/z, in which hydroxyl group remains. The remaining fragments at m/z 222.0219, and 151.0457 represent minor fragments formed during the degradation of the ß-lactam ring, in which the amide group from the aliphatic chain is retained.

In the MS/MS spectrum (Figure S5) of TP1 recorded in negative ion mode the first and most prominent peak appears at 464.0770 m/z. This fragment results from the cleavage at the C8 carbon and the attached carbonyl oxygen alongside with the removal of attached hydroxyl group at C6. The peak at 420.0475 m/z represents descarbamoyl cefuroxime axetil with the lost methyl moiety within axetil group. Fragmentation of this structure then leads to formation of a lactone structure at 378.0395 m/z. This fragment readily loses attached hydroxyl group, giving rise to an ion at 360.0284 m/z. Alternatively, a fragment with an m/z of 329.0676 is formed, as a result of the loss of a hydroxyl group and a sulfur atom from the ß-lactam ring. Additionally, decarboxylation of the lactone structure leads to peak with m/z of 334.0470, or a more intensive peak at 316.0396 m/z, corresponding to the further loss of a hydroxyl group. A minor fragment at 416.1106 m/z represents structure after partial degradation of axetil moiety, detachment of acetamide and cleavage of the C8 carbon and the attached carbonyl oxygen in the ß-lactam ring. The fragment with an m/z of 420.0475 can also undergo further fragmentation to produce a fragment with an m/z of 257.0293. Following the loss of a sulfur atom and an attached hydroxyl group, this ion is then converted into a fragment with an m/z of 207.0370. A

small fragment resulting from the cleavage of the ß-lactam ring, along with an amide group that is part of the aliphatic chain, observed as peak with m/z of 148.0101. Additional peaks representing structures with a partially degraded ß-lactam ring and an attached hydroxyl group are observed at 312.0652 and 276.0613. Notably, the m/z 276.0613 retains the hydroxyl group despite the loss of sulfur. The peak with an m/z of 60.0099 represents acetamide.

In the MS/MS spectrum (Figure S6) of TP2 recorded in positive ion mode, fragmentation begins with the detachment of the axetil moiety (peak at 463.0519 m/z). This fragment undergoes subsequent decarboxylation, forming an ion with an m/z of 419.0632. Notably, a prominent fragment corresponding to a lactone structure is also observed at 402.0351 m/z, which is further decarboxylated to generate an ion at 358.0465 m/z. Analogous to TP1, the presence of the fragment with an m/z of 291.0026 suggests the possible presence of a hydroxyl group attached either to a sulfur atom or to the C6 or C7 positions within the ß-lactam ring. The presence of fragments at 265.0261 m/z and 231.0354 m/z clearly rules out any modifications within the furan ring of TP2. Partial degradation of the ß-lactam ring leads to the formation of product ions with m/z of 195.0355 and 134.0207, both of which retain an attached oxygen atom. During the fragmentation process, it is noteworthy that the attached oxygen remains strongly associated with the ß-lactam ring and is only eliminated during the formation of the fragment with 195.0355 m/z, where it is lost together with the sulfur atom.

In the MS/MS spectrum (Figure S7) recorded in negative ion mode, degradation begins with the removal of axetil moiety, first through the detachment of the acetyl group (481.0699 m/z), followed by the loss of an oxygen atom (465.0767 m/z). The formation of a lactone structure results in the most prominent peak in this spectrum, observed at 378.0403 m/z. This lactone fragment then undergoes a minor loss of an oxygen atom forming a peak at 360.0341 m/z. Decarboxylation of these structures leads to the formation of ions 334.0525 m/z and 316.0434 m/z. Alternatively, further degradation of the 481.0699 m/z fragment leads to the formation of ions with m/z of 421.0460 or 393.0451. The cleavage of ß-lactam ring leads to a minor peaks with m/z of 290.0626, 137.0195, 106.0552, and 92.0186. Finally, the peak at m/z 60.0096 corresponds to acetamide.

The fragmentation of the third transformation product, similar to the two previous products, begins with the removal of the axetil fragment. In the MS/MS spectrum (Figure S8) of TP3 recorded in positive ion mode, a peak at 523.0750 m/z is observed, which undergoes further degradation to a structure with m/z of 481.0630. This corresponds to cefuroxime with two hydroxyl groups substituted in place of the double bond within ß-lactam ring. Direct evidence for this is provided by the fragment at 393.0834, which corresponds to a fragment observed in the parent spectrum at m/z of 359.0795, with the hydroxyl group intact, formed after the removal of (furan-2-yl)(methoxyimino)acetaldehyde. The fragment at 481.0630 m/z undergoes decarboxylation to a lesser extent, yielding a fragment with an m/z of 437.0708, and to a greater extent to a structure with an m/z of 376.0566 m/z. Loss of one oxygen atom as water is then observed in the peak at 358.0427 m/z. Cleavage at the C8 carbon and the attached carbonyl oxygen within ß-lactam ring leads to a peak with m/z of 332.0642. Alternatively, fragmentation of the ion with an m/z of 481.0630 leads to the formation of ions with m/z of 283.0334, and 265.0422, where the carboxyl group at C4 in the ß-lactam ring is retained. The absence of the fragments at 134.0178 m/z and 291.0041 m/z, which are visible in the spectra in the positive mode of the two monooxygenated products, or analogous structures, suggests that oxidation did not occur at positions C6, C7, or the sulfur atom. A small peak at 133.0356 m/z results from the degradation of the ß-lactam ring and cyclization with remaining amide group in the aliphatic chain.

In the MS/MS spectrum (Figure S9) recorded in negative ion mode, present is prominent peak at 464.0810 m/z corresponding to descarbamoyl cefuroxime axetil. Ion with the m/z of 439.0616 m/z represents structure after axetil detachment and loss of one attached hydroxyl group. Formation of a lactone structure is observed at 396.0523 m/z with the present two attached hydroxyl groups. One of them is readily loss and a fragment with the m/z of 378.0415 is formed, and the loss of second group leads to the formation of 360.0303 m/z fragment. Further loss of carbonyl leads to the formation of a peak with m/z of 334.0373. The degradation can also lead to the formation of a structure represented by a peak with m/z of 233.0375.

In the case of TP4 with m/z 191.0427, it was not possible to obtain an MS/MS spectrum. The structure was determined based on the exact recorded mass, supported by the observation of a short retention time and the absence of isomers during chromatographic separation.

TP5 and TP6 are chlororganic products. In the case of TP5 the fragmentation begins from the acetyl removal within axetil moiety, represented in its MS/MS spectrum (Figure S10) by a peak with an m/z of 539.0251 m/z. A visible is prominent peak at m/z value of 497.0151 (Figure S11), representing cefuroxime after detachment of axetil, with retained hydroxyl group and chlorine atom. Formation of a lactone structure with retained hydroxyl group is observed (400.0229 m/z). At this point, the chlorine atom has already been cleaved off together with a proton. From this structure either carbonyl can be detached (peak at 374.0356 m/z), either carbon dioxide (peak at 356.0283 m/z). Two fragments resulted from ß-lactam ring degradation, 291.0036 m/z and 231.0410 m/z, together clearly ruled out the possibility of the changes within furan ring. Moreover, the presence of the fragments with m/z of 291.0036 and 185.0023 suggests that oxidation could only occur at

positions C6, C7, or the sulfur atom of the ß-lactam ring. The presence of the fragment with an m/z of 200.0202 (Figure S12) indicates the presence of attached hydroxyl group at position C6 of the ß-lactam ring.

In the case of TP6 fragmentation, the process also begins with the removal of the axetil moiety. In its MS/MS spectrum (Figure S13), peaks with m/z values of 539.0284 and 497.0148 correspond to the removal of the acetyl group and the complete axetil moiety, respectively. Alternatively, loss of the chlorine atom may occur with the formation of the peak at 547.0767 m/z. Peaks with m/z value of 400.0260 represents a lactone structure (Figure S14). The loss of chlorine is observed, but the mass suggests that the additional oxygen remains. The fragments resulted from ß-lactam ring degradation with an m/z value of 291.0072 remains the additional oxygen atom. However, considering the presence of the fragment represented by the peak at 255.9859 m/z, which is formed after partial degradation of ß-lactam ring and contains both additional oxygen and chlorine atoms, it suggests that the oxygen atom is positioned at C6, C7, or with sulfur atom. The fragment with an m/z value of 182.9930 (Figure S15) represent a fragment resulted from the cleavage of ß-lactam ring and provides direct evidence for the presence of the chlorine atom at position 6 of the β-lactam ring. Based on the observation that the additional oxygen atom persists in the fragmentation spectra for an extended period and is only lost in conjunction with the sulfur atom, it can be assumed that this species is likely a sulfoxide.

TP7 is a pair of anti-cefuroxime axetil diastereoisomers, and its MS/MS spectrum (Figure S16) is analogous to the spectrum of the parent molecule. The fragmentation begins with the removal of the axetil moiety, initially partially (489.0647 m/z) and eventually entirely (447.0563 m/z). Following the detachment of acetamide, a lactone if formed (386.0420 m/z), and after the loss of carbon dioxide, a peak with m/z of 342.0490 appears. The removal of methoxy group results in a peak with m/z of 314.0561. The spectrum also shows a peak at 359.0769 m/z, which corresponds to the detachment of (furan-2-yl)(methoxyimino)acetaldehyde.

TP7, under acidic conditions, is oxidize to form the TP8 and TP9, which are analogous to the structures of TP1 and TP2. TP8 is the anti-isomer of TP1. In its MS/MS spectrum (Figure S17), fragmentation begins with the removal of axetil group (463.0567 m/z), which is further converted into the lactone structure (402.0346 m/z). Following the loss of carbon dioxide, a peak at 358.0488 m/z is formed. The final peak results from the removal of methoxy group (peak at 330.0516 m/z). All of these fragments are similar to these observed in the MS/MS spectrum of TP1. In the MS/MS spectrum of TP9 (Figure S18), fragmentation begins similarly to the parent spectrum, with the removal of the axetil moiety (463.0503 m/z). As with TP8, further fragmentation leads to the formation of lactone structure (402.0360 m/z), followed by the loss of carbon dioxide (358.0441 m/z). All observed fragments in the spectra of TP8 and TP9 are analogous to those in the parent spectrum, but in each fragment, the attached hydroxyl group is retained.

In the MS/MS spectrum of TP10 (Figure S19), corresponding to cefuroxime, all of the present fragment ions are also present in the spectrum of cefuroxime axetil. Notably, no fragments indicative of the axetil group are detected, suggesting that fragmentation originates from the core structure after the loss of the axetil moiety. The fragmentation process begins with the formation of a peak at 403.0606 m/z, representing a loss of single oxygen atom from the carboxyl group. Similarly to the parent spectrum, a distinct peak at m/z 386.0416 is observed, which resulted from detachment of acetamide with the cyclization to well-characterized lactone structure. Further fragmentation leads to degradation of ß-lactam ring, resulting in a peak with m/z of 231.0392. There is also visible a peak at 175.0009 m/z, which arises from the partial degradation of the ß-lactam ring followed by intramolecular cyclization.

Table S1. Parameters of River water and Lake water.

Parameter	River Water	Lake Water
pH	7.8	7.2
Conductivity	553 µS/cm	152 µS/cm
NPOC (mg/L)	3.363	22.09
Chlorides (mg/L)	112	7
Nitrates (mg/L)	5.06	1.69
Sulphates (mg/L)	58	5.4
Phosphates (mg/L)	0.177	<0.05

Table S2. LC-MS and TOC parameters.

Device	Parameter	Value	
LC	Solvents	A – acetonitrile	
		B – 0.1% aqueous solution of HCOOH	
	Gradient	5% A to 60% A	
	Analysis time	9 min	
	Post-time equilibration	2 min	
	Flow rate	0.3 mL min^{-1}	
	Injection volume	2 μL	
	Column temperature	35 °C	
MS	Ion source	Electrospray (ESI)	
	Mode	Positive	Negative
	Source temperature	325 °C	325 °C
	Drying gas flow	11 L min^{-1}	10 L min^{-1}
	Nebulizer pressure	50 psig	40 psig
	Capillary voltage	3500 V	3500 V
	Fragmentor voltage	175 V	175 V
	Skimmer voltage	65 V	65 V
	Octopole voltage	750 V	750 V
	Mass range	70 - 950 m/z	70 – 1050 m/z
	Acquisition rate	2.0 spectra s^{-1}	2.0 spectra s^{-1}
TOC	Measurement method	NPOC	
	Injection volume	1 mL	
	Automatic dilution	10	
	Number of washes	1	
	Carrier/sparge gas	Synthetic air	
	Sparge gas flow	80 mL min^{-1}	
	Sparge time	1.5 min	
	Acid (HCl) addition	1.5%	

Table S3. Accurate masses of the TPs of cefuroxime axetil formed during electrolysis in negative mode.

Name	t_R	Mass [m/z]		Error [ppm]	Elemental formula	Fragmentation (MS/MS)	
		Theoret.	Experim			Mass [m/z]	Elemental formula
Cefuroxime axetil	5.9, 6.05	509.0984	509.0990	1.18	$C_{20}H_{21}N_4O_{10}S$	466.0904	$C_{19}H_{20}N_3O_9S$
						421.0772	$C_{16}H_{13}N_4O_8S$
						405.0503	$C_{16}H_{13}N_4O_7S$
						378.0753	$C_{16}H_{16}N_3O_6S$
						362.0459	$C_{15}H_{12}N_3O_6S$
						318.0557	$C_{14}H_{12}N_3O_4S$
						284.0700	$C_{11}H_{14}N_3O_4S$
						251.0152	$C_{10}H_7N_2O_4S$
						207.0412	$C_9H_7N_2O_4$
						193.0063	$C_9H_9N_2O_3S$
						116.0350	$C_4H_6NO_3$
						92.0143	C_5H_2NO
						60.0109	CH_2NO_2
TP1	4.8, 4.9	525.0933	525.0939	1.14	$C_{20}H_{21}N_4O_{11}S$	464.0734	$C_{19}H_{18}N_3O_9S$
						420.0475	$C_{17}H_{14}N_3O_8S$
						416.1106	$C_{16}H_{22}N_3O_8S$
						378.0368	$C_{15}H_{22}N_3O_7S$
						360.0327	$C_{15}H_{10}N_3O_6S$
						343.0290	$C_{12}H_{11}N_2O_8S$
						334.0470	$C_{14}H_{12}N_3O_5S$
						329.0676	$C_{15}H_{11}N_3O_6$
						316.0348	$C_{14}H_{10}N_3O_4S$
						312.0652	$C_{12}H_{14}N_3O_5S$
						276.0613	$C_{12}H_{10}N_3O_5$
						257.0293	$C_9H_9N_2O_5S$
						207.0370	$C_9H_7N_2O_4$
						148.0102	$C_4H_6NO_3S$
						60.0099	CH_2NO_2
TP2	5.1, 5.2	525.0933	525.0952	3.62	$C_{20}H_{21}N_4O_{11}S$	481.0613	$C_{18}H_{17}N_4O_{10}S$
						465.0767	$C_{18}H_{17}N_4O_9S$
						421.0429	$C_{16}H_{13}N_4O_8S$
						378.0403	$C_{15}H_{12}N_3O_7S$
						360.0297	$C_{15}H_{10}N_3O_6S$
						334.0525	$C_{14}H_{12}N_3O_5S$
						316.0410	$C_{14}H_{10}N_3O_4S$
						300.0594	$C_{11}H_{14}N_3O_5S$
						137.0170	$C_6H_5N_2S$
						106.0552	$C_3H_{10}N_2S$
						92.0186	C_5H_2NO
						60.0105	CH_2NO_2
TP3	3.6	543.1039	543.1056	3.13	$C_{20}H_{23}N_4O_{12}S$	464.0780	$C_{19}H_{18}N_3O_9S$
						439.0504	$C_{16}H_{15}N_4O_9S$
						396.0544	$C_{15}H_{15}N_3O_8S$
						378.0350	$C_{15}H_{12}N_3O_7S$
						360.0282	$C_{15}H_{10}N_3O_6S$
						334.0493	$C_{14}H_{12}N_3O_4S$
						223.0344	$C_9H_7N_2O_5S$

Table S4. NPOC data in conducted experiments

Experiment	Time (min)	NPOC (mg/L)	Average NPOC (mg/L)	C/C_0	Average C/C_0	SD
pH3	0	6.558	6.082	1	1	0
		5.606		1		
	15	6.289	5.831	0.9590	0.9587	0.000385

		5.373		0.9584		
	30	6.403	5.697	0.9764	0.9367	0.060734
		4.992		0.8905		
	60	5.192	4.704	0.7917	0.7734	0.027913
		4.217		0.7522		
	90	4.494	4.098	0.6853	0.6738	0.017485
		3.703		0.6605		
	120	3.907	3.521	0.5958	0.5789	0,025837
		3.135		0.5592		
pH7	0	6.909	6.231	1	1	0
		5.552		1		
	15	6.313	5.798	0.913736	0.9305	0.026738
		5.283		0.951549		
	30	5.979	5.500	0.865393	0.8827	0.027553
		5.021		0.904359		
	60	5.471	4.972	0.791866	0.79794	0.009751
		4.473		0.805656		
	90	4.999	4.400	0.723549	0.7061	0.027656
		3.800		0.684438		
	120	4.181	3.521	0.605153	0.5651	0.063529
		2.861		0.51531		
pH9	0	6.279	5.969	1	1	0
		5.659		1		
	15	6.538	5.845	1.041249	0.9792	0.092393
		5.153		0.910585		
	30	5.917	5.436	0.942348	0.9107	0.047325
		4.954		0.87542		
	60	5.208	4.584	0.829431	0.7680	0.091684
		3.960		0.69977		
	90	4.191	3.759	0.667463	0.6298	0.056125
		3.328		0.58809		
	120	3.816	3.120	0.60774	0.5227	0.126727
		2.425		0.428521		
RW	0	9.635	8.950	1	1	0
		8.265		1		
	15	8.262	7.836	0.857499	0.8755	0.0277
		7.411		0.896673		
	30	7.618	7.205	0.790659	0.8050	0.02209
		6.793		0.8219		
	60	5.583	5.123	0.57945	0.5724	0.013616
		4.630		0.560194		
	90	4.171	3.666	0.432901	0.4096	0.035755
		3.160		0.382335		
	120	3.064	2.701	0.318007	0.3018	0.24839
		2.338		0.8288		
LW	0	24.920	24.450	1	1	0
		23.980		1		
	15	22.300	21.640	0.894864	0.8851	0.013825
		20.990		0.875313		
	30	19.560	18.990	0.784912	0.7767	0.011564
		18.430		0.768557		
	60	15.070	14.530	0.604735	0.5943	0.015379
		13.980		0.582986		
	90	11.350	10.870	0.455457	0.4446	0.015389
		10.400		0.433695		
	120	8.164	7.6610	0.327608	0.3133	0.020584
		7.158		0.298499		
Chlorides	0	6.210	5.905	1	1	0

50 mg L^{-1}		5.600		1		
	15	6.102	5.661	0982609	0.9587	0.035558
		5.221		0.932321		
	30	5.797	5.328	0.933494	0.9024	0.046539
		4.859		0.867679		
	60	4.800	4.302	0.772947	0.7285	0.066355
		3.803		0.679107		
	90	3.901	3.440	0.62818	0.5826	0.068161
		2.978		0.531786		
	120	3.143	2.767	0.506119	0.4686	0.055971
		2.391		0.426964		
Chlorides 100 mg L^{-1}	0	5.905	5.508	1	1	0
		5.111		1		
	15	5.892	5.413	0.997798	0.9828	0.02307
		4.933		0.965173		
	30	5.499	4.916	0.931245	0.8925	0.059157
		4.332		0.847584		
	60	4.700	4.128	0.795936	0.7495	0.070839
		3.556		0.695754		
	90	3.813	3.269	0.645724	0.5935	0.079592
		2.725		0.533164		
	120	2.873	2.421	0.486537	0.4395	0.071622
		1.969		0.385248		
Humic Acid 10 mg L^{-1}	0	9.354	9.072	1	1	0
		8.791		1		
	15	9.308	8.749	0.995082	0.9644	0.044945
		8.189		0.931521		
	30	9.200	8.617	0.983536	0.9498	0.049328
		8.033		0.913775		
	60	7.906	7.451	0.8452	0.8213	0.034841
		6.997		0.795928		
	90	7.323	6.662	0.782874	0.7343	0.070883
		6.001		0.68263		
	120	6.375	5.901	0.681527	0.6505	0.04539
		5.427		0.617336		
Humic Acid 20 mg L^{-1}	0	13.370	13.090	1	1	0
		12.810		1		
	15	13.210	12.740	0.988033	0.9733	0.02189777
		12.260		0.957065		
	30	12.550	12.040	0.938669	0.9198	0.02673568
		11.540		0.900859		
	60	11.600	10.960	0.867614	0.8373	0.04438799
		10.310		0.80484		
	90	10.680	9.918	0.798803	0.7577	0.05970775
		9.151		0.714364		
	120	9.234	8.515	0.690651	0.6505	0.05797258
		7.797		0.608665		
Fulvic Acid 10 mg L^{-1}	0	9.797	9.557	1	1	0
		9.316		1		
	15	9.898	9.441	1.0103093	0.9879	0.032489
		8.984		0.9643624		
	30	9.342	8.891	0.9535572	0.9303	0.03365
		8.440		0.9059682		
	60	8.269	7.834	0.844034	0.8197	0.035296
		7.398		0.7941176		
	90	7.314	6.763	0.746555	0.7076	0.056388
		6.212		0.66681		
	120	7.360	6.216	0.7512504	0.6504	0.146313

		5.071		0.544332		
Fulvic Acid	0	13.700	13.500	1	1	0
20 mg L^{-1}		13.230		1		
	15	13.150	12.590	0.9598540	0.9326	0.036284
		12.020		0.9085412		
	30	12.930	12.220	0.9437956	0.9052	0.052187
		11.510		0.8699924		
	60	11.330	10.610	0.8270072	0.7859	0.05634976
		9.887		0.747317		
	90	9.504	8.924	0.693723	0.6610	0.04451889
		8.345		0.630763		
	120	8.145	7.556	0.594526	0.5597	0.04802623
		6.967		0.526606		
NOM	0	9.463	8.954	1	1	0
10 mg L^{-1}		8.445		1		
	15	9.212	8.638	0.9734756	0.9647	0.01314589
		8.064		0.9548845		
	30	9.047	8.392	0.9560393	0.9372	0.02811279
		7.738		0.9162818		
	60	7.707	7.200	0.8144352	0.8041	0.01548225
		6.693		0.79254		
	90	6.643	6.142	0.7019973	0.6860	0.023978
		5.642		0.6680876		
	120	5.962	5.358	0.6300328	0.5984	0.047444
		4.754		0.5629366		
NOM	0	13.360	12.960	1	1	0
20 mg L^{-1}		12.550		1		
	15	12.910	12.330	0.9663174	0.9514	0.02125732
		11.750		0.9362550		
	30	12.370	11.770	0.9258982	0.9082	0.02479226
		11.180		0.8908367		
	60	10.820	10.280	0.8098802	0.7932	0.02366396
		9.744		0.7764143		
	90	9.421	8.928	0.7051647	0.6889	0.02331574
		8.436		0.6721912		
	120	8.262	7.682	0.6184132	0.5927	0.03713496
		7.102		0.565896		

Table S5. Aquatic toxicity of cefuroxime axetil and its TPs [mg L^{-1}]

Cpd.	Fish Acute LC50 IRFMN	Fish Chronic NOEC IRFMN	F. minnow LC50 96h EPA	Algae Acute EC50 IRFMN	Algae Chronic NOEC IRFMN
Cef. ax.	6.37	0.4115	18.31	7.45	1.38
TP1	6.47	0.4261	10.91	7.41	1.44
TP2	6.51	0.3844	39.14	16.17	0.96
TP3	6.7	0.4252	51.82	6.57	1.86
TP4	28.16	2.11	274.53	28.5	3.06
TP5	7.11	0.3435	3.53	8.57	0.7628
TP6	7.31	0.3701	14.05	8.31	0.8959
TP7	6.37	0.4115	18.31	7.45	1.38
TP8	6.47	0.4261	10.91	7.41	1.44
TP9	6.51	0.3844	39.14	16.17	0.96
TP10	6.12	0.1805	33.46	6.02	0.5175

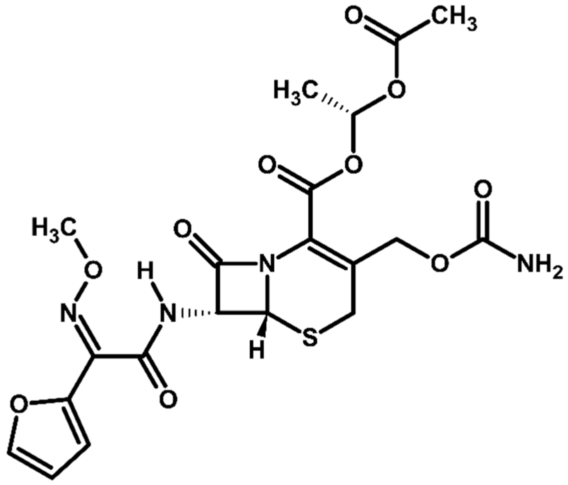

Cefuroxime axetil diastereoisomer A

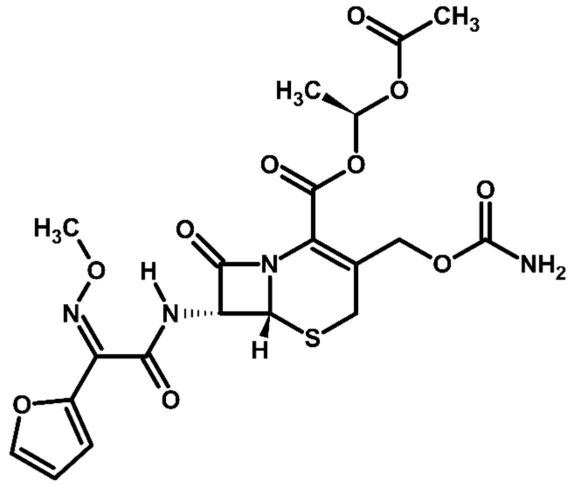

Cefuroxime axetil diastereoisomer B

Figure S1. The structures of diastereoisomers A and B of cefuroxime axetil, with retention times of 6.05 and 5.90 minutes, respectively, using the applied method.

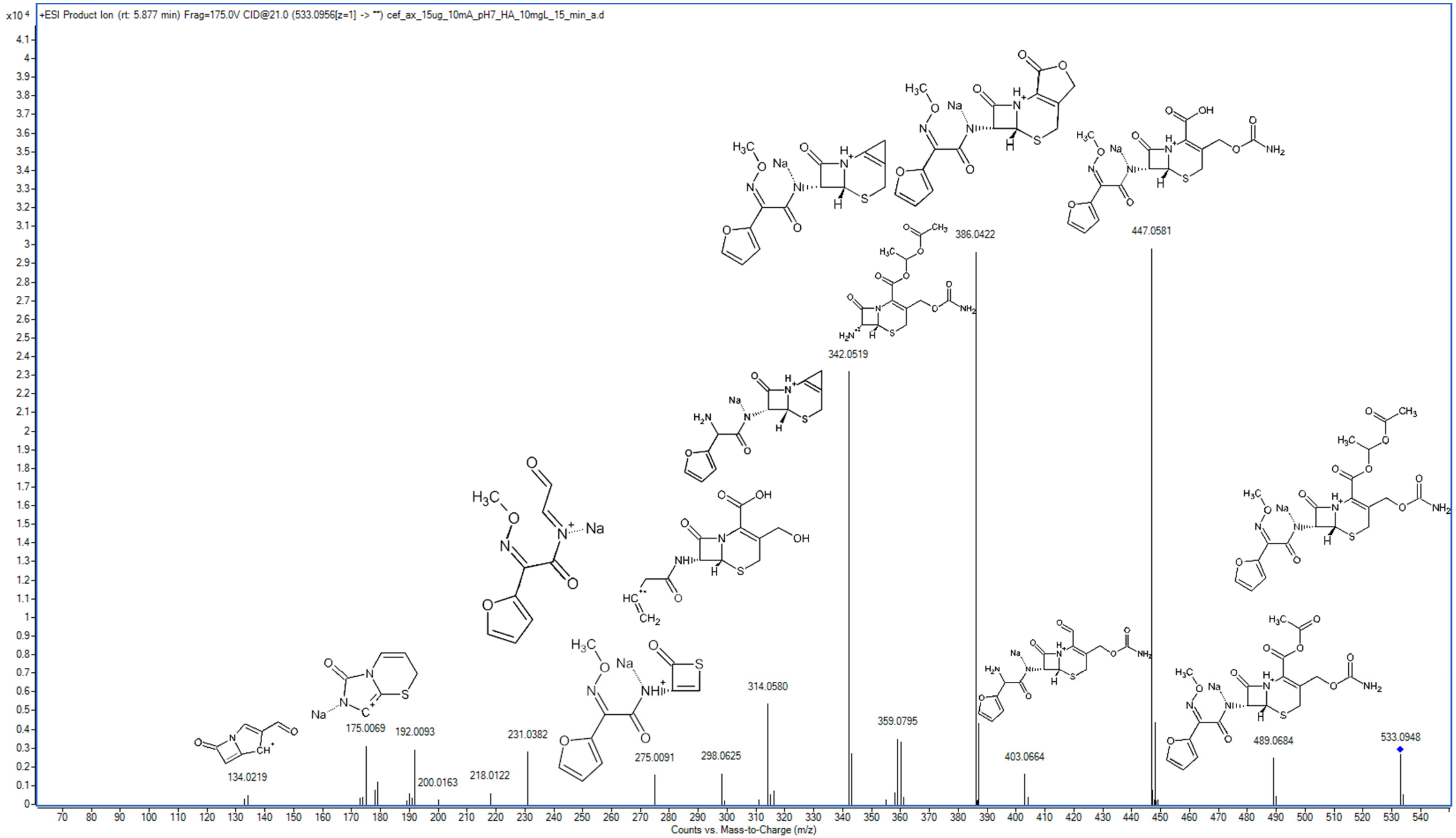

Figure S2. MS/MS spectrum and fragmentation pattern of cefuroxime axetil in positive ion mode.

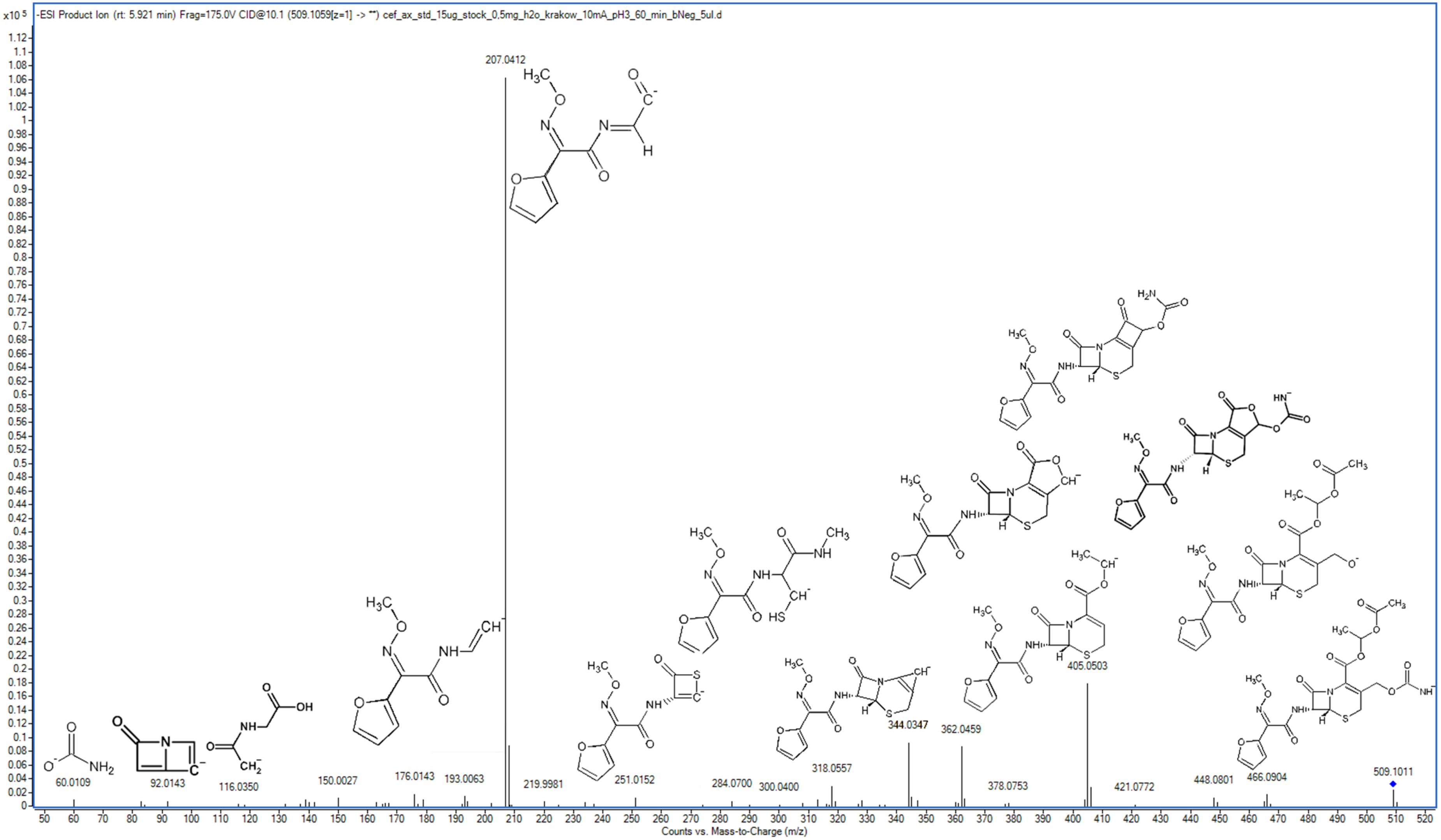

Figure S3. MS/MS spectrum and fragmentation pattern of cefuroxime axetil in negative ion mode.

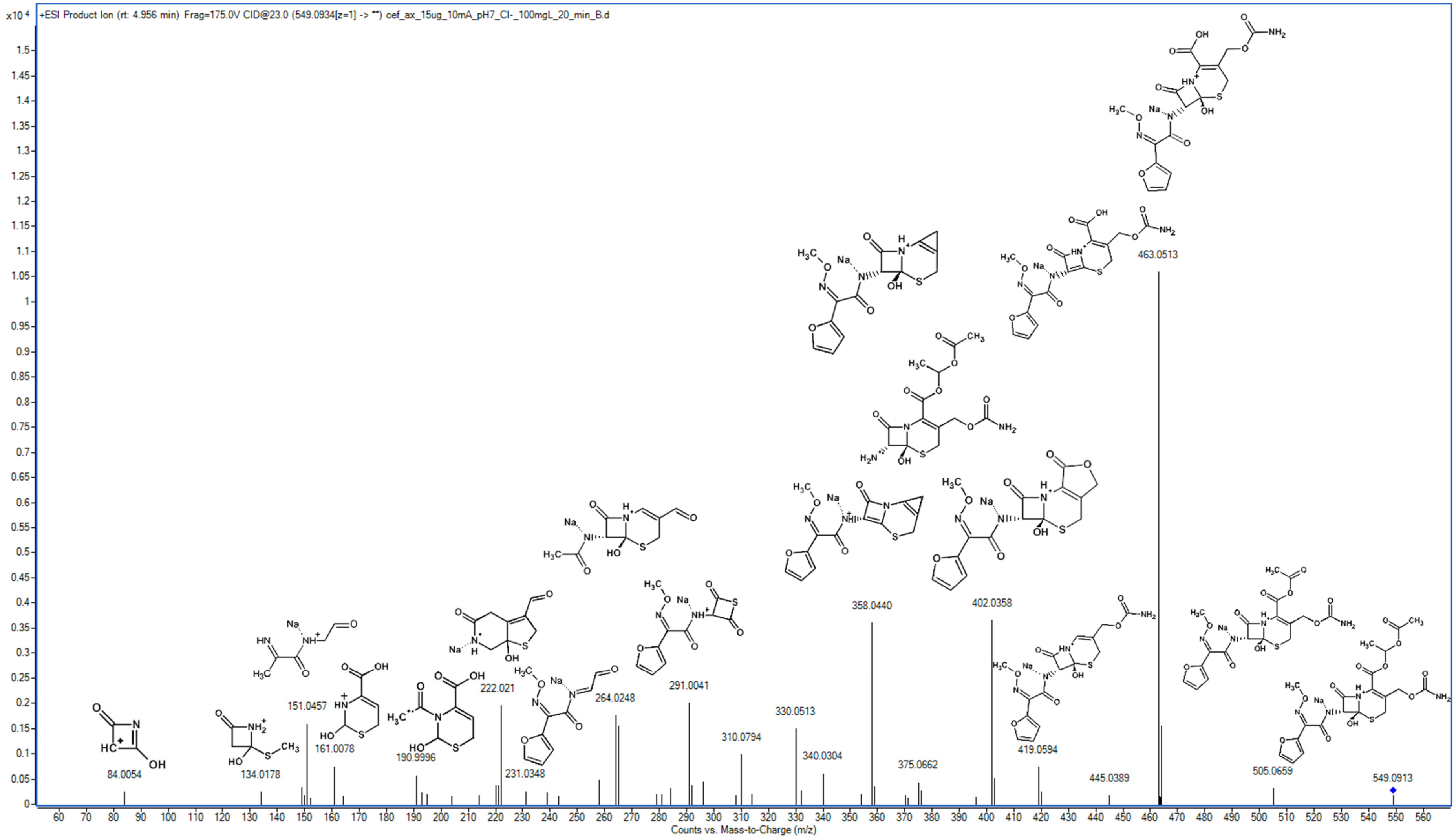

Figure S4. MS/MS spectrum and fragmentation pattern of TP1 in positive ion mode.

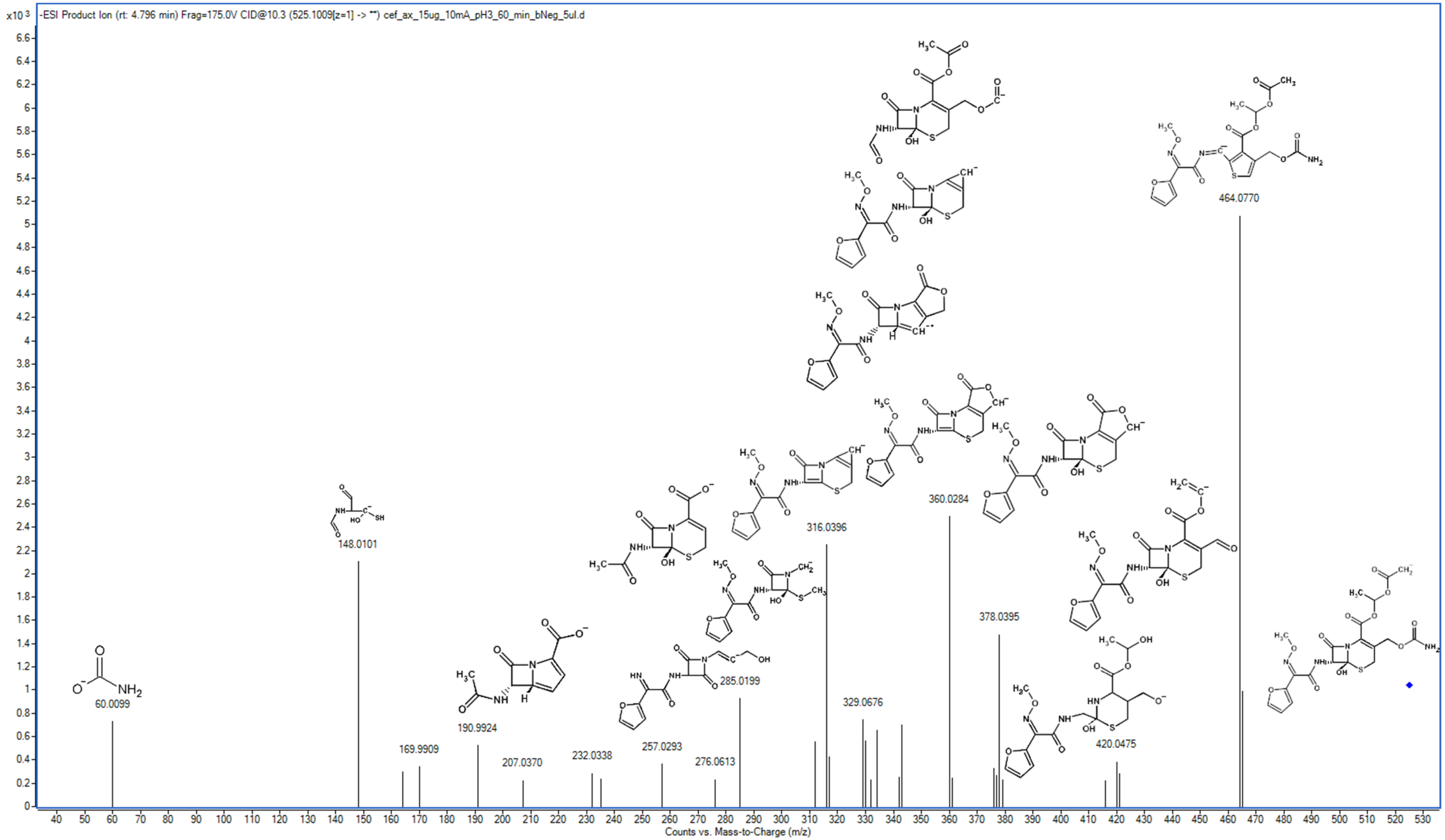

Figure S5. MS/MS spectrum and fragmentation pattern of TP1 in negative ion mode.

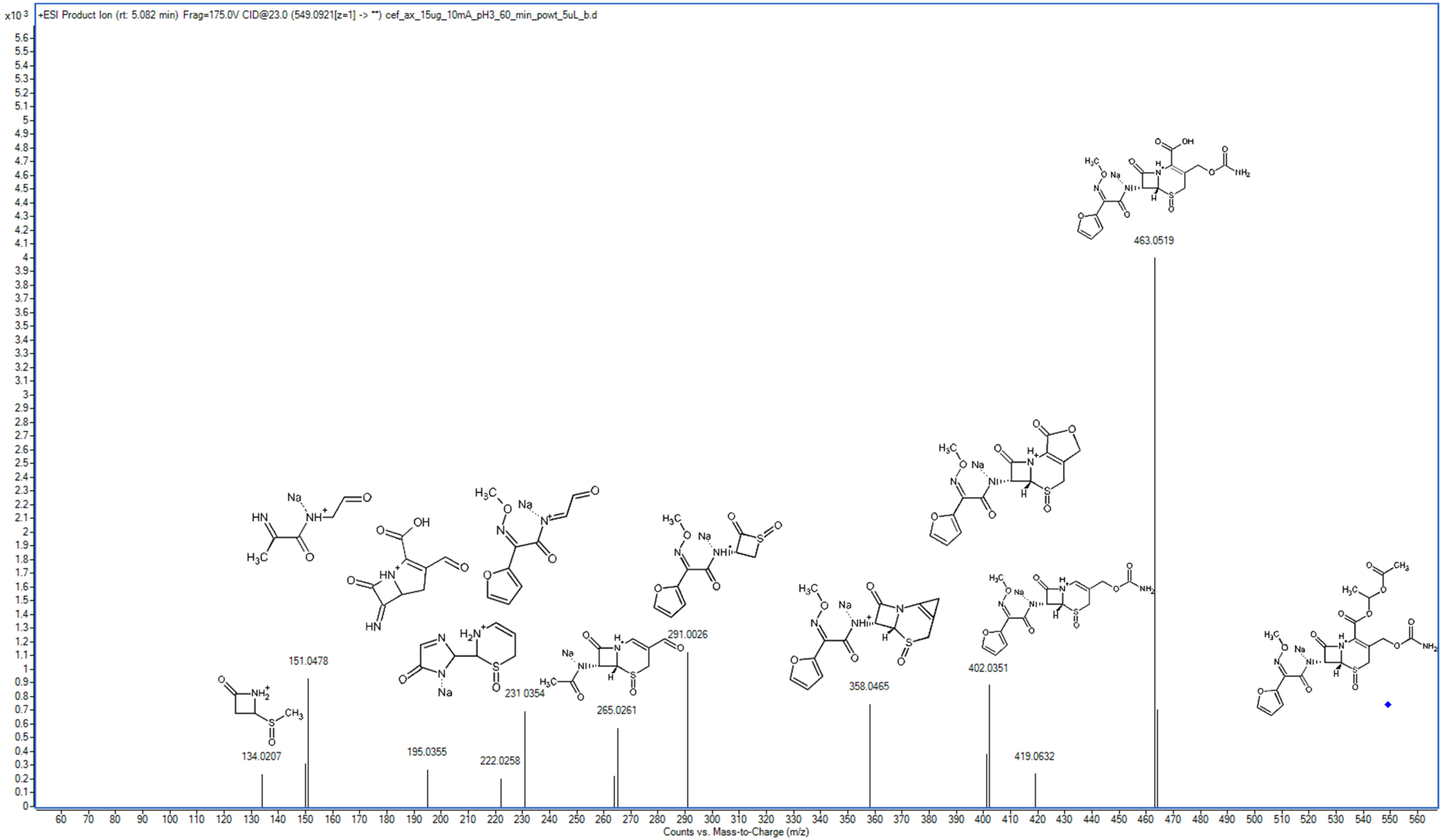

Figure S6. MS/MS spectrum and fragmentation pattern of TP2 in positive ion mode.

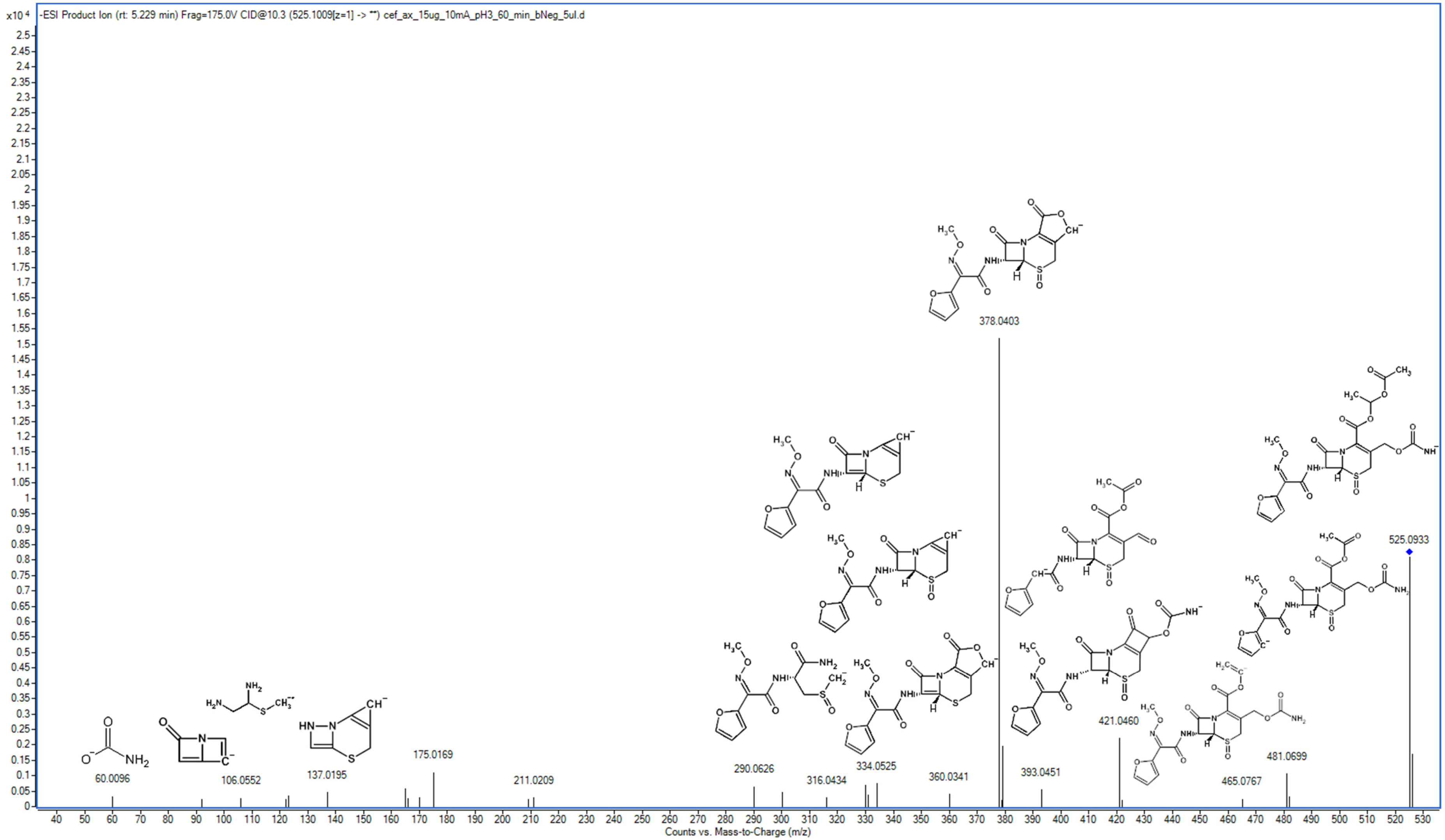

Figure S7. MS/MS spectrum and fragmentation pattern of TP2 in negative ion mode.

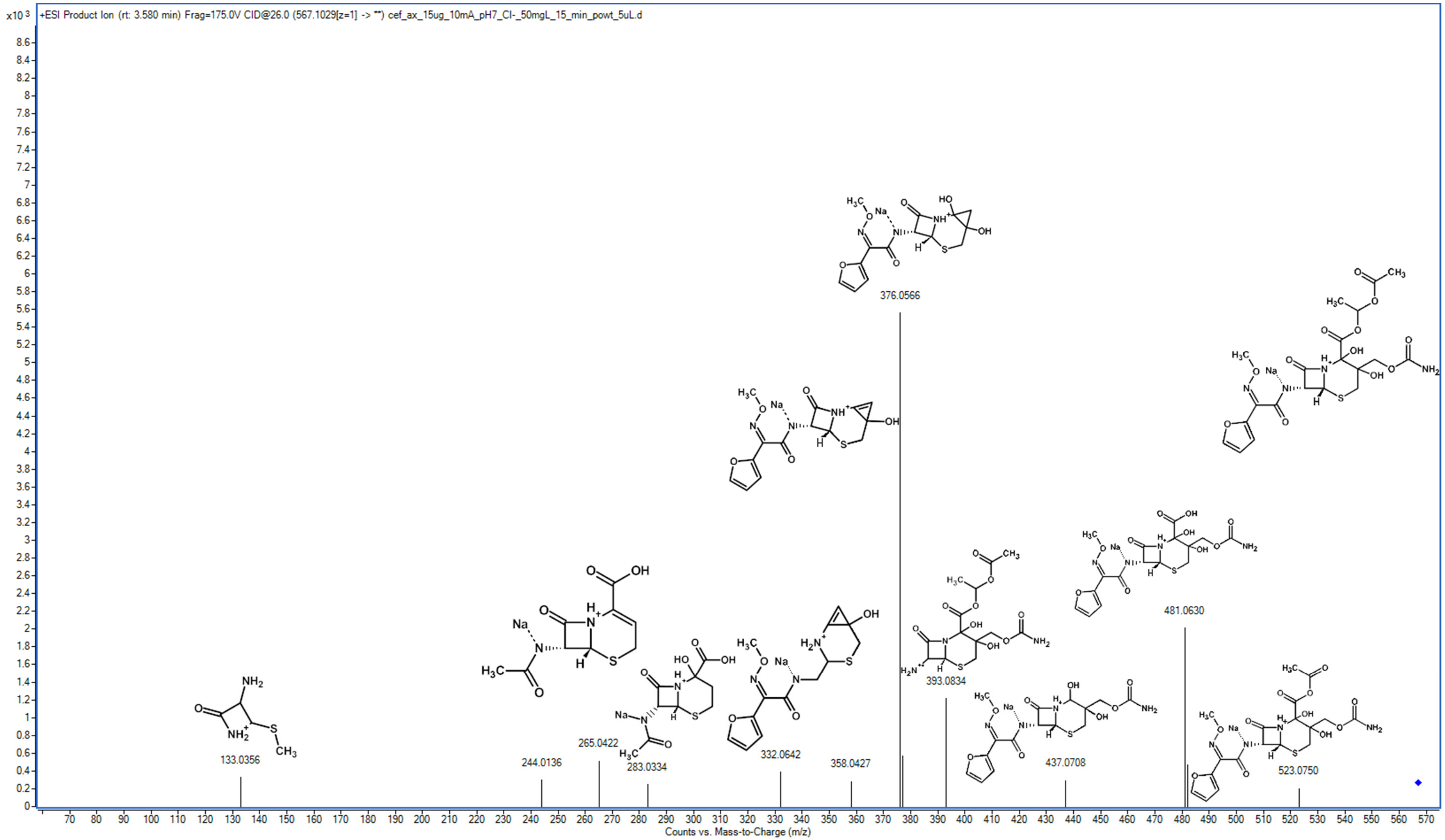

Figure S8. MS/MS spectrum and fragmentation pattern of TP3 in positive ion mode.

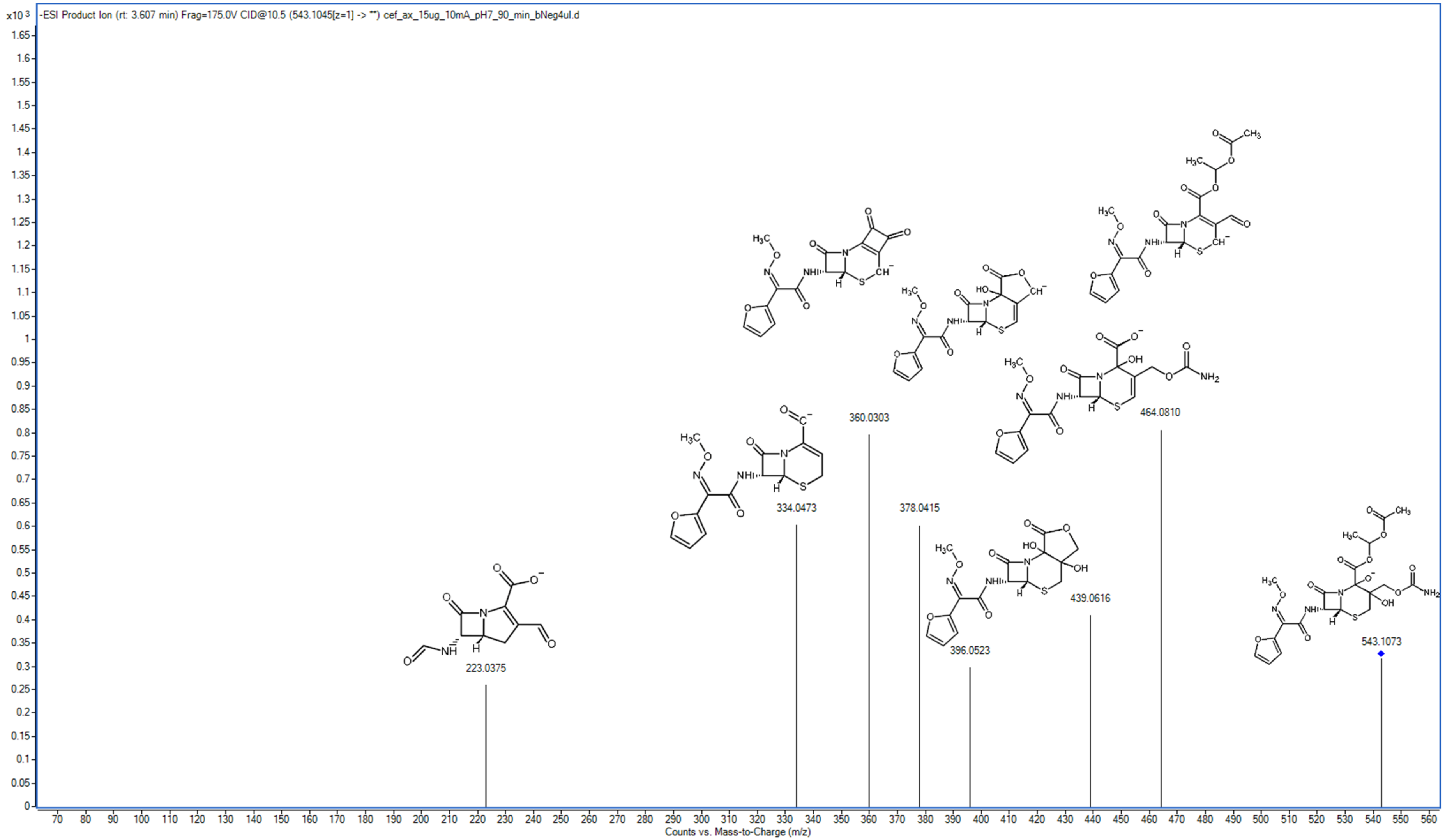

Figure S9. MS/MS spectrum and fragmentation pattern of TP3 in negative ion mode.

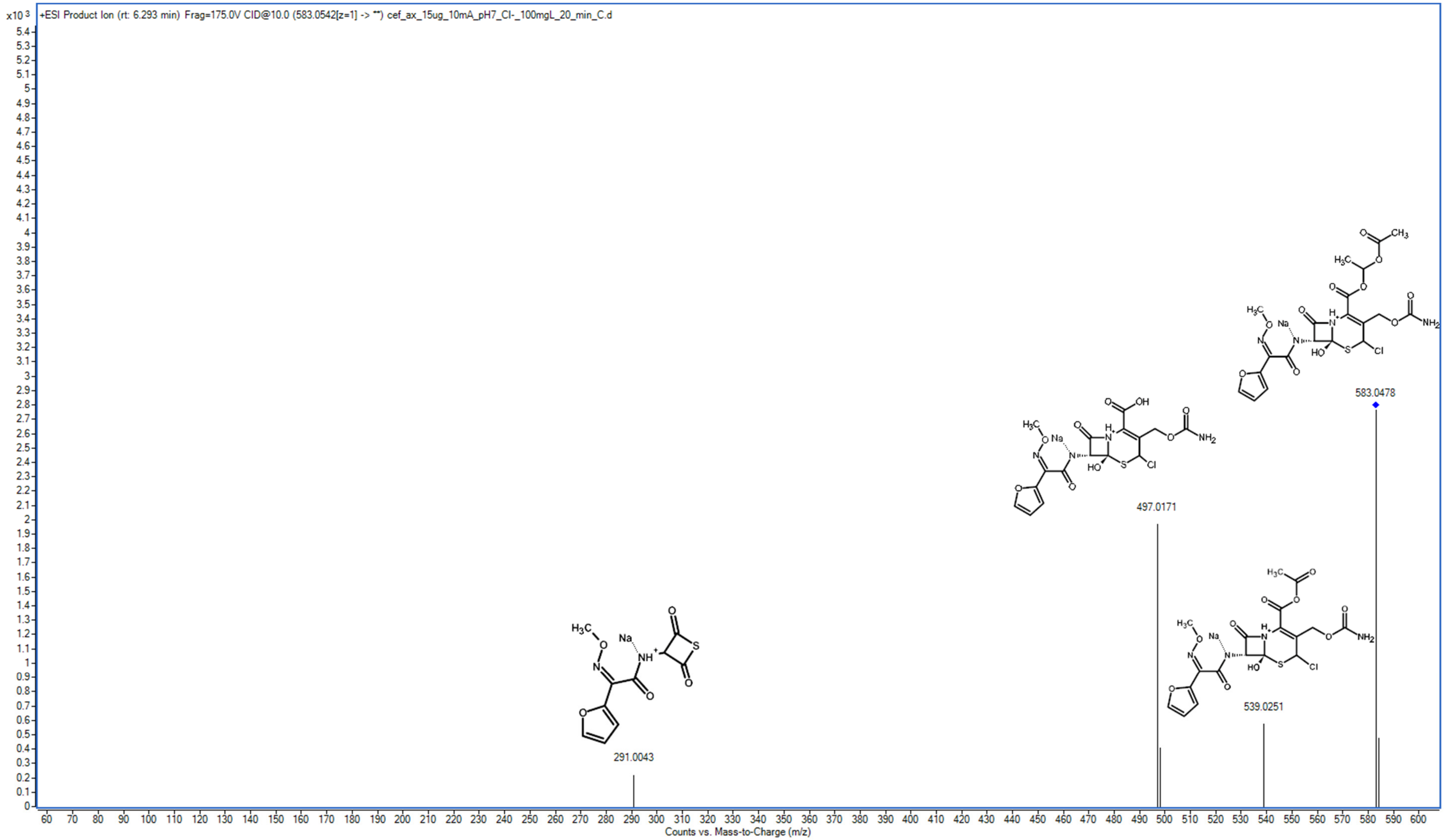

Figure S10. MS/MS spectrum and fragmentation pattern of TP5 in positive ion mode (part 1).

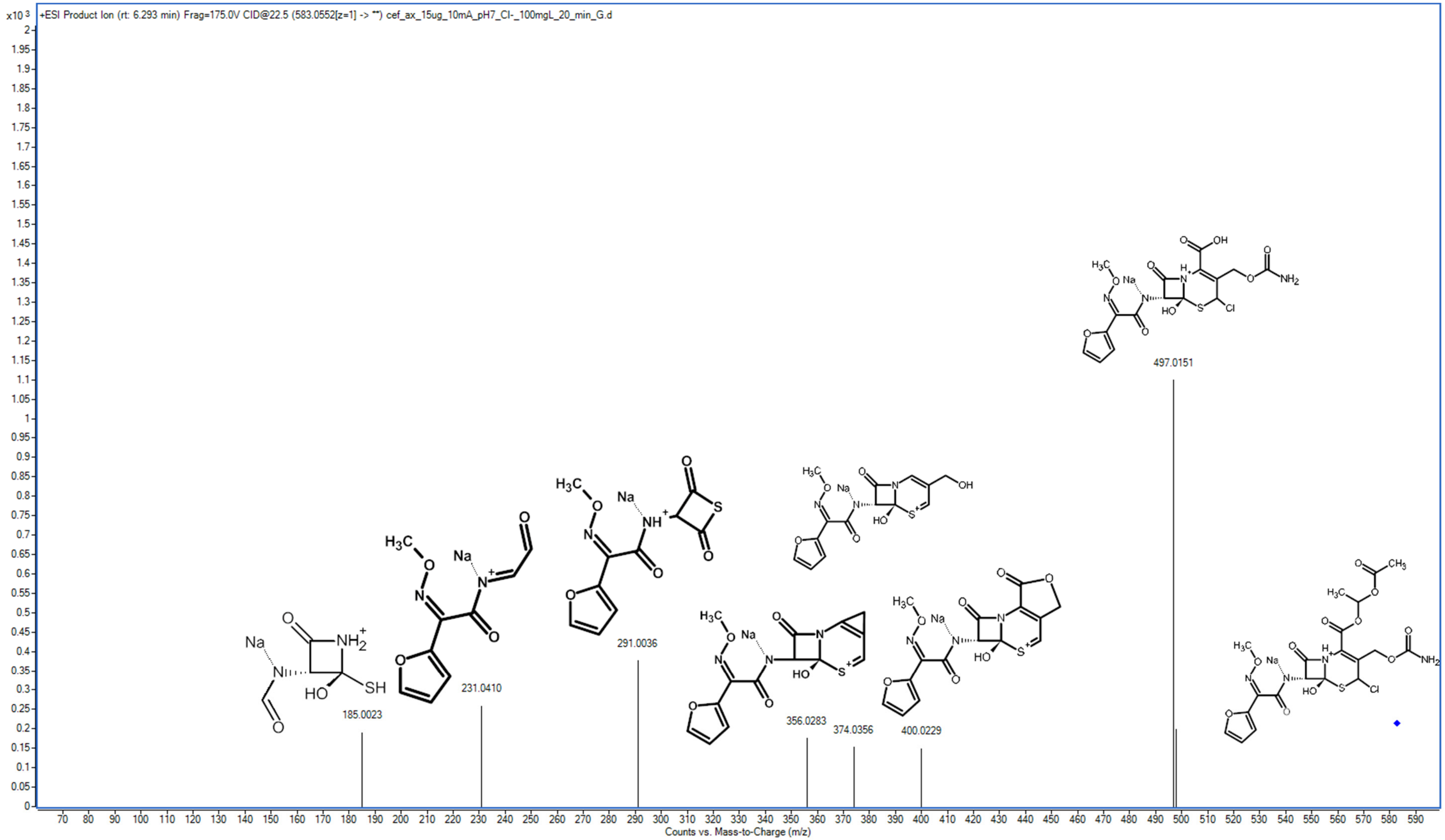

Figure S11. MS/MS spectrum and fragmentation pattern of TP5 in positive ion mode (part 2).

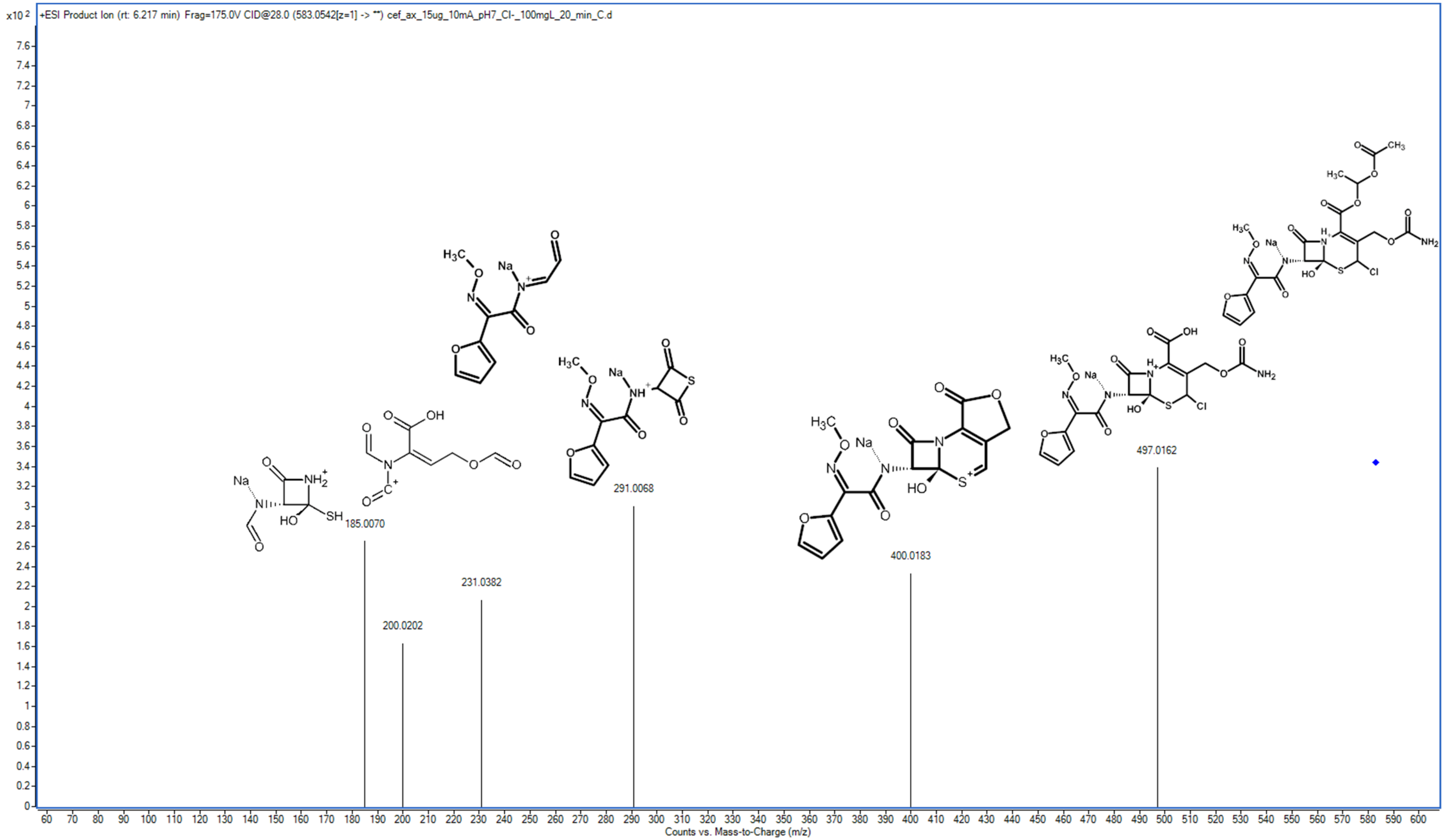

Figure S12. MS/MS spectrum and fragmentation pattern of TP5 in positive ion mode (part 3).

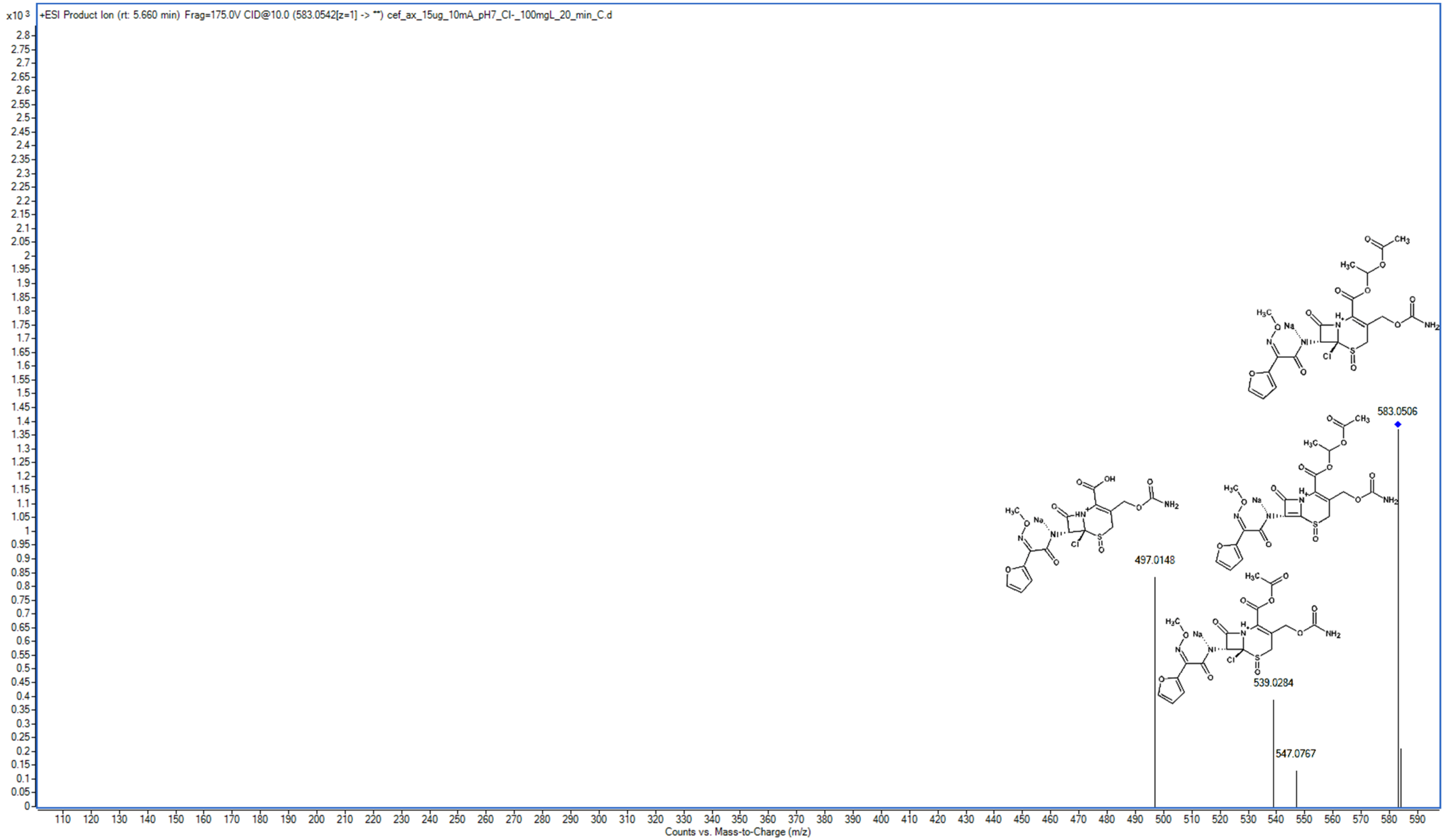

Figure S13. MS/MS spectrum and fragmentation pattern of TP6 in positive ion mode (part 1).

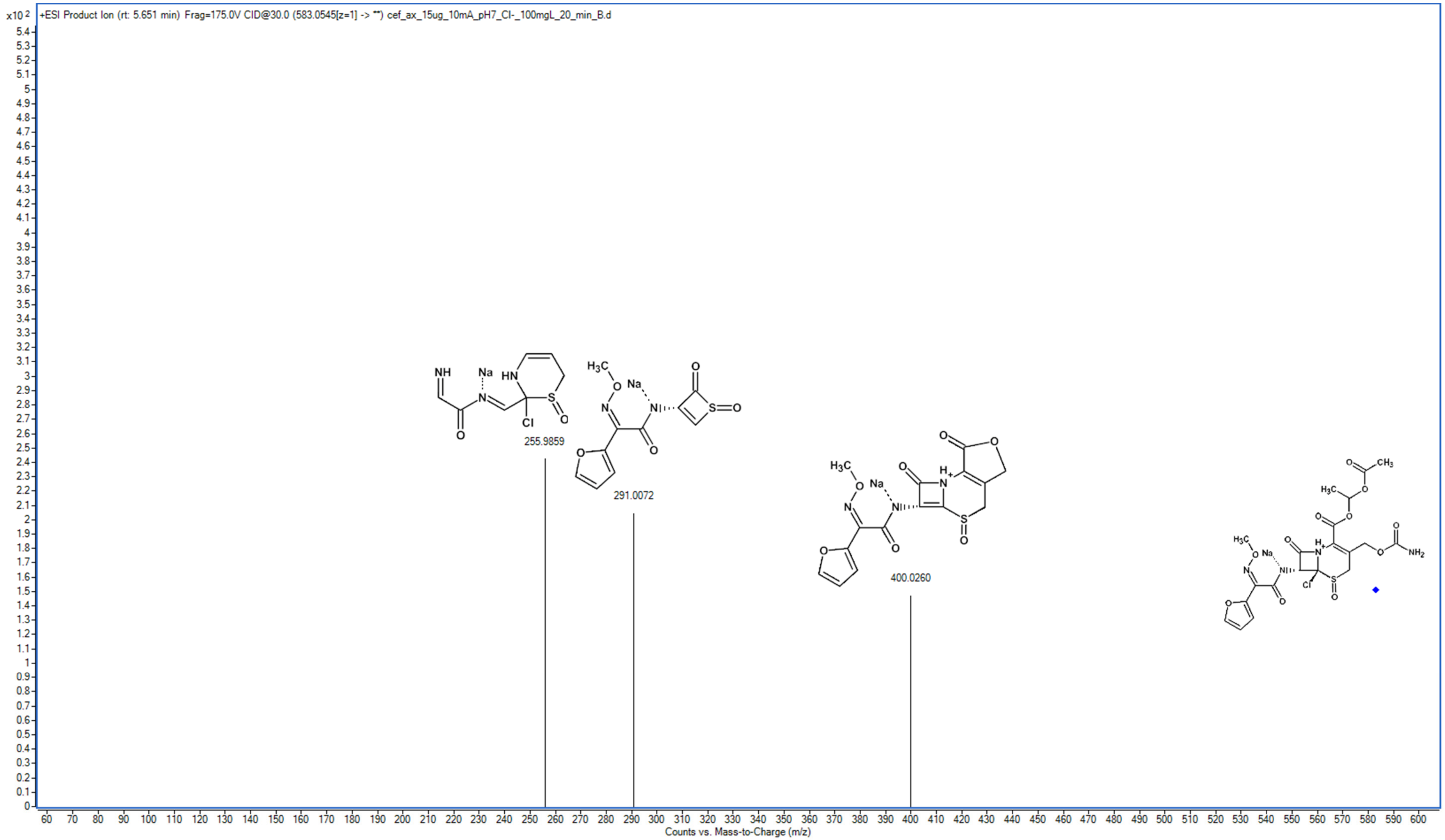

Figure S14. MS/MS spectrum and fragmentation pattern of TP6 in positive ion mode (part 2).

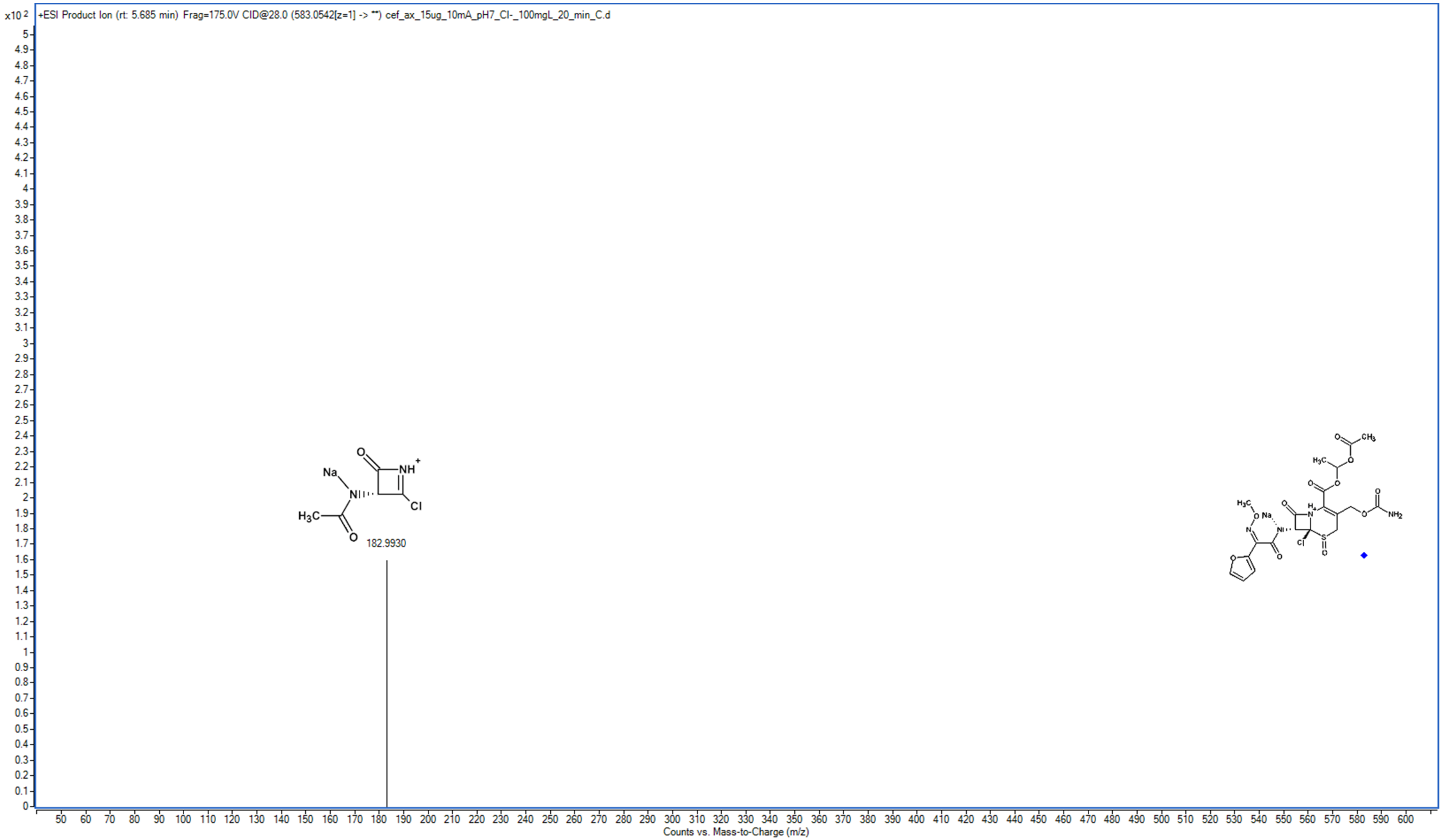

Figure S15. MS/MS spectrum and fragmentation pattern of TP6 in positive ion mode (part 3).

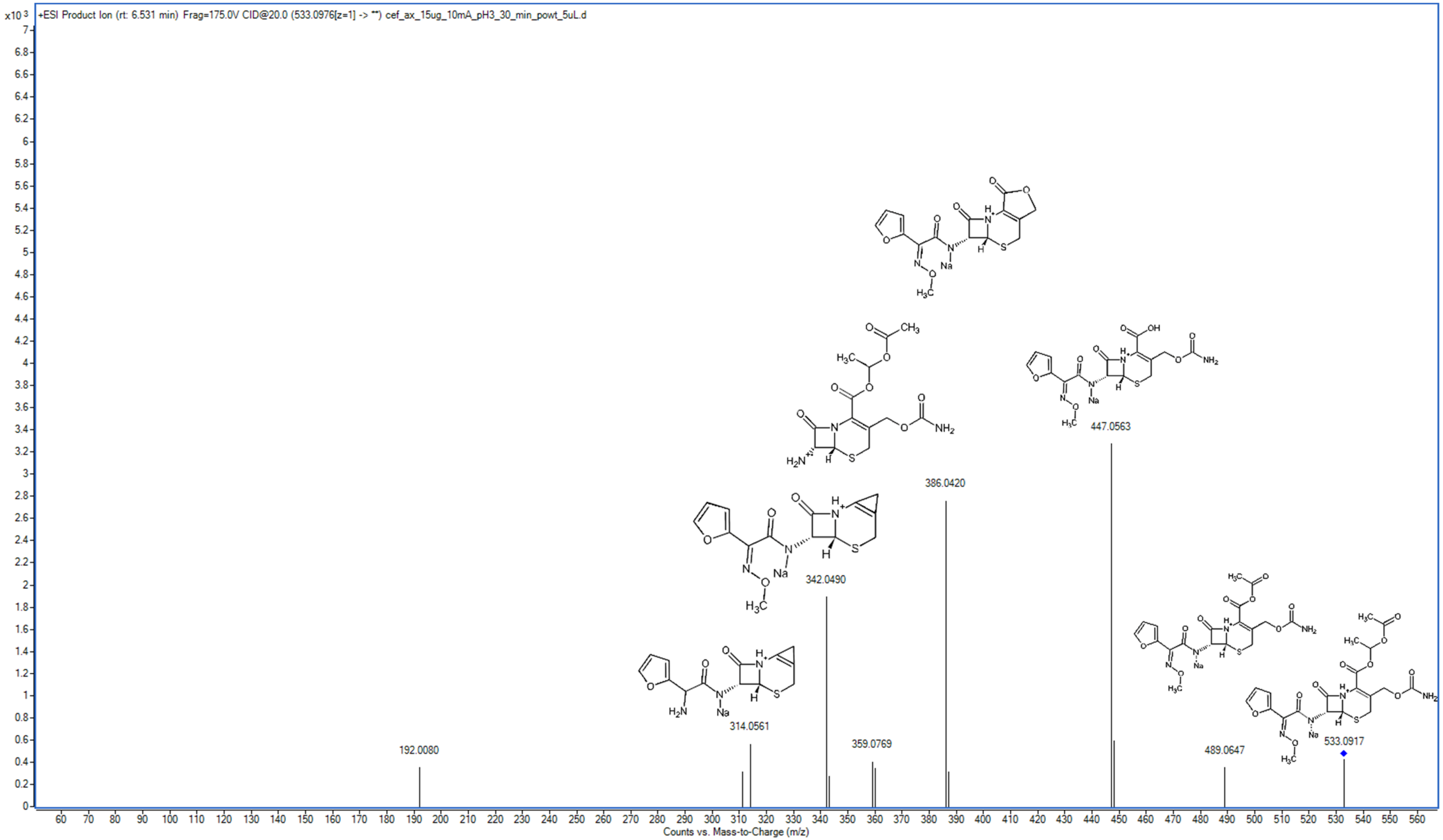

Figure S16. MS/MS spectrum and fragmentation pattern of TP7 in positive ion mode.

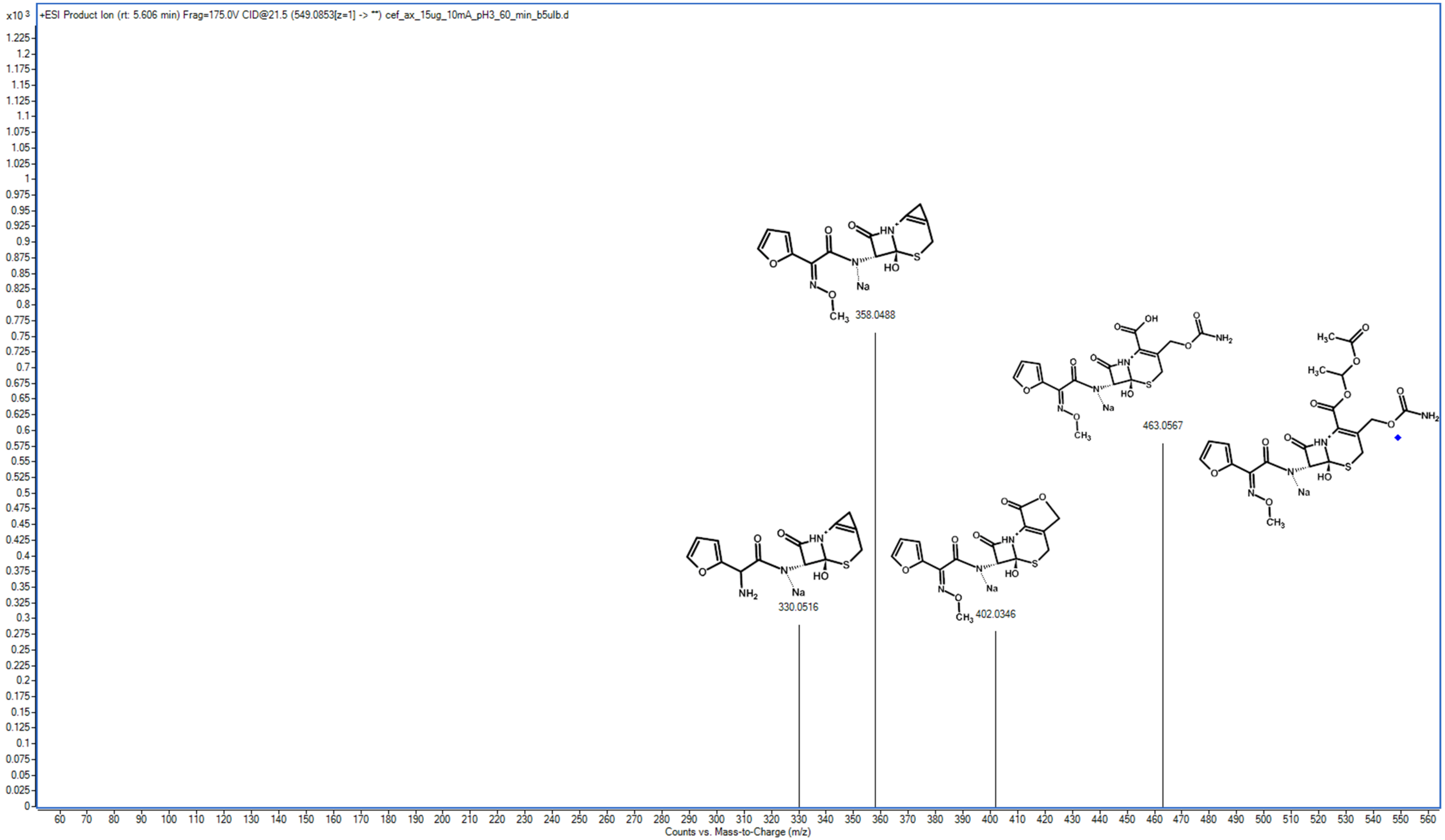

Figure S17. MS/MS spectrum and fragmentation pattern of TP8 in positive ion mode.

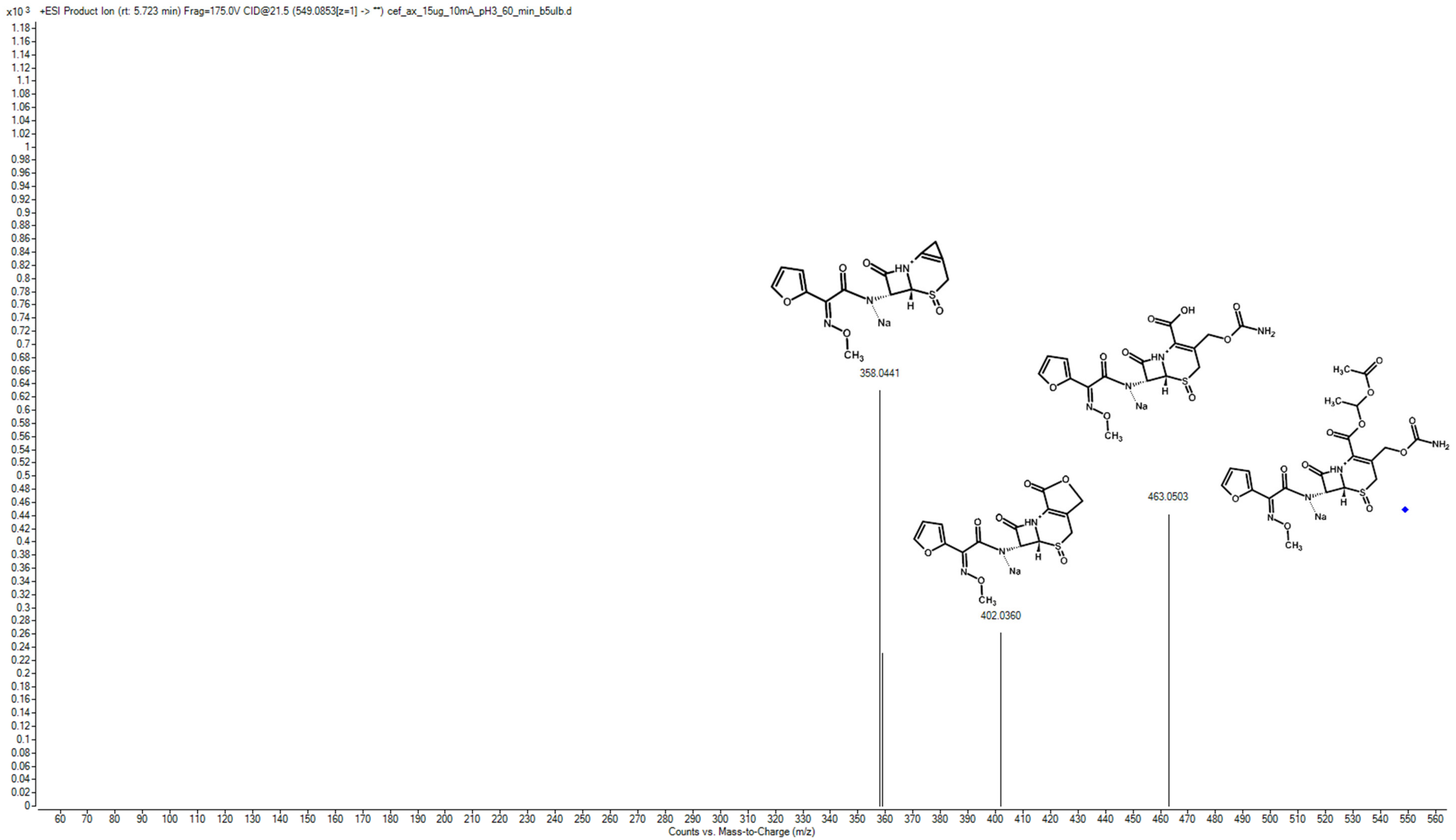

Figure S18. MS/MS spectrum and fragmentation pattern of TP9 in positive ion mode.

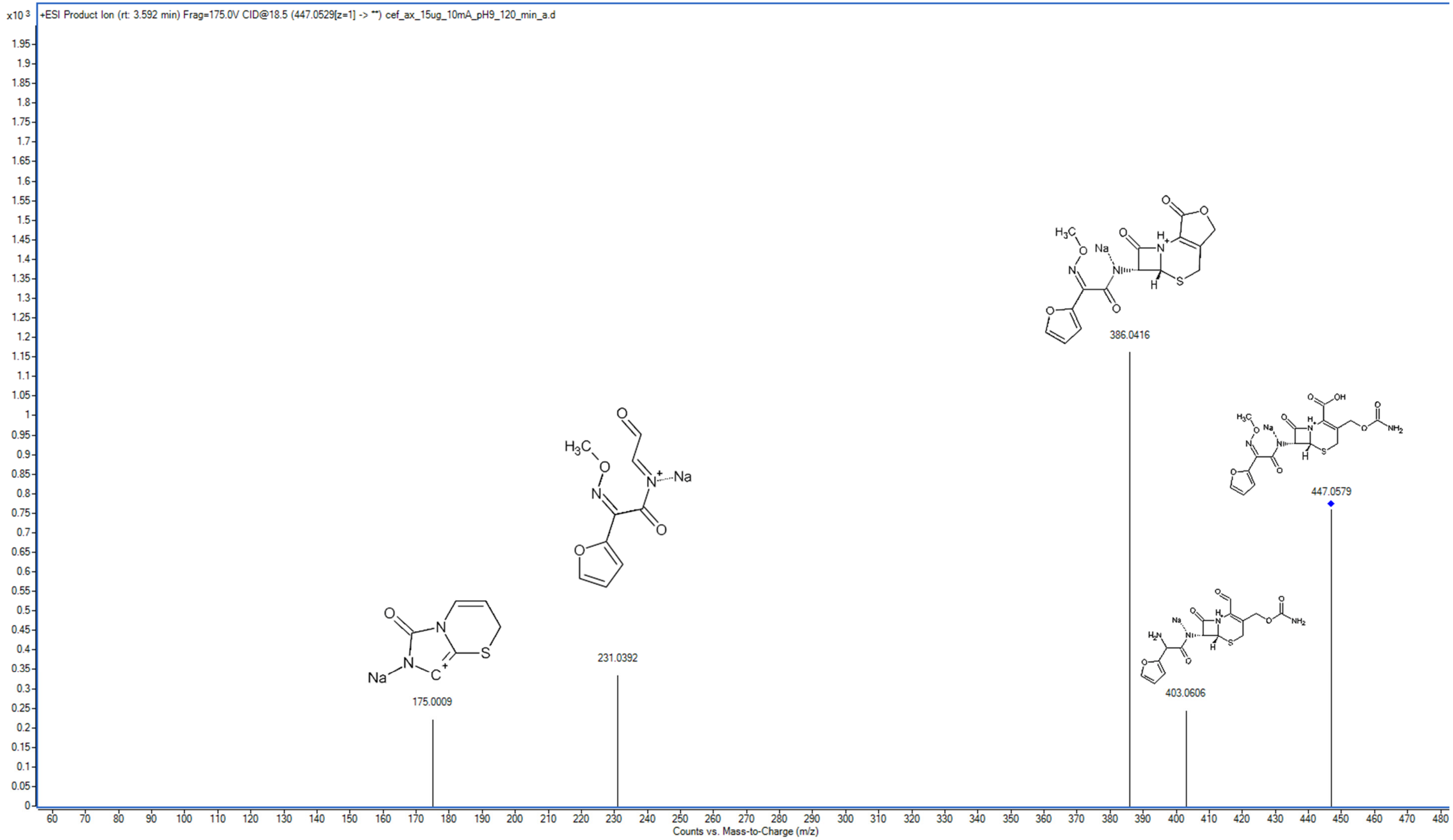

Figure S19. MS/MS spectrum and fragmentation pattern of TP10 in positive ion mode.
